# Neural Efficiency in Athletes: A Systematic Review

**DOI:** 10.3389/fnbeh.2021.698555

**Published:** 2021-08-05

**Authors:** Longxi Li, Daniel M. Smith

**Affiliations:** Department of Physical Education and Health Education, Springfield College, Springfield, MA, United States

**Keywords:** neural efficiency, athletes, sports, neuroimaging, neuroscience

## Abstract

According to the neural efficiency hypothesis (NEH), professionals have more effective cortical functions in cognitive tasks. This study is focusing on providing a systematic review of sport-related NEH studies with functional neuroimaging or brain stimulation while performing a sport-specific task, with the aim to answer the question: How does long-term specialized training change an athlete's brain and improve efficiency? A total of 28 studies (*N* = 829, Experimental Group *n* = 430) from 2001 to 2020 (Median = 2014, *SD* = 5.43) were analyzed and results were organized into four different sections: expert-novice samples, perceptual-cognitive tasks and neuroimaging technologies, efficiency paradox, and the cluster analysis. Researchers examined a wide range of sport-specific videos and multiple object tracking (MOT) specific to 18 different sports and utilized blood oxygenation-level dependent (BOLD) functional magnetic resonance imaging (fMRI), functional near-infrared spectroscopy (fNIRS), and electroencephalogram (EEG). Expert-novice comparisons were often adopted into investigations about the variations in general about optimal-controlled performance, neurophysiology, and behavioral brain research. Experts tended to perform at faster speeds, more accurate motor behavior, and with greater efficiency than novices. Experts report lower activity levels in the sensory and motor cortex with less energy expenditure, experts will possibly be more productive. These findings generally supported the NEH across the studies reviewed. However, an efficiency paradox and proficient brain functioning were revealed as the complementary hypothesis of the NEH. The discussion concentrates on strengths and key limitations. The conclusion highlights additional concerns and recommendations for prospective researchers aiming to investigate a broader range of populations and sports.

## Introduction

“As increasing levels of expertise are attained, there are measurable changes in neural activation” (Vickers and Williams, 2017, p. 5). Historically, the neural efficiency hypothesis (NEH) “was first proposed by Haier et al. (1988), who adopted positron emission tomography (PET) to determine the relationship between task performance and level of neural activation during the performance of intelligence tests” (Vickers and Williams, 2017, p. 5). Haier et al. found “an inverse relationship between brain glucose metabolism levels and the score obtained on the intelligence test” (Vickers and Williams, 2017, p. 5). Participants who “had high intelligence scores consumed less energy than those with lower scores and performed more quickly, leading the authors to suggest that superior intelligence was due to neural circuits that performed at faster speeds and with greater efficiency” (Vickers and Williams, 2017, p. 5). In general, neural efficiency consists of better performance during the repetition of a task (Babiloni et al., 2009), lower energy consumption in completing same performance (Zhang et al., 2019), and relatively less pronounced alpha ERD as a commonly used index of neural efficiency or spatially selective cortical activation (Del Percio et al., 2008; Babiloni et al., 2010). Higher neural efficiency is characterized by a bidirectional reduction phenomenon encompassing both reduced activation of areas associated with task execution and reduced deactivation of regions associated with irrelevant information processing (Qiu et al., 2019).

In the last decades, several pieces of evidence extended the NEH to the cortical motor and visual systems of “expert athletes” such as elite kendo and gymnasts (Kita et al., 2001), elite rifle and gun shooters (Fattapposta et al., 1996; Haufler et al., 2000; Janelle et al., 2000; Loze et al., 2001; Di Russo et al., 2005; Del Percio et al., 2008), and elite karate and fencing athletes (Del Percio et al., 2009a,b). In recent years, progressive research was conducted in the neuroscience field to interpret athletes' performances. Callan and Naito (2014) addressed four neural mechanisms that might primarily contribute to experts' exceeding performance over novices: neural efficiency, cortical expansion, specialized processes, and internal models. These four neural mechanisms are correlated to revealing evidence about the expert and novice athletes' differences and similarities in brain activity. Recently, Filho et al. (2021) suggested complementary neural mechanisms of neural efficiency, neural proficiency, and transient hypertonicity theoretically correlated with optimal performance in experts.

In this way, investigators in the domain of motor learning suggested that “skilled performance was defined by high levels of automaticity, minimum energy expenditure, and reduced movement times” (Schmidt and Lee, 2014; Vickers and Williams, 2017, p. 5). According to Vickers and Williams (2017), “these documented changes have led to a general ‘faster-is-better’ approach in terms of defining optimal motor behavior, brain function, and assumptions about how athletes should be trained” (p. 5). For instance, athletes are often instructed to “shift their gaze rapidly and accelerate their thought processes and movements to the point of reducing the level of conscious control of what they are doing” (Shepherd, 2015; Vickers and Williams, 2017, p. 5). However, reducing conscious control did not lead directly to the non-conscious processes. Although robust evidence was found through non-conscious contributions to action control were strong, the non-conscious and conscious action control were still not fully understood (Shepherd, 2015). Moreover, this runs counter to the literature on quiet eye (QE), which calls for the performer to maintain their visual focus and concentration on a specific location during a critical final phase of movement (Vickers, 2016). Formally, QE is defined as “the final fixation or tracking gaze that is located on a specific location or object in the task environment within 3° of visual angle (or less) for a minimum of 100 ms” (Vickers, 2016, p. 119). In a comprehensive review of intelligence and the NEH, “Neubauer and Fink (2009) reported 29 studies in support of the hypothesis, while 18 provided mixed support and nine had contradictory results” (Vickers and Williams, 2017, p. 5). According to Neubauer and Fink (2009), a possible reason for the contradictory results is the variability in task difficulty across the studies they reviewed. That is, some studies incorporated tasks that may not have been demanding enough to find support for the NEH.

In addition, Neubauer and Fink (2009) concluded that the neural efficiency was mostly observed for low-to-moderately difficult tasks and in the frontal lobe of the brain. However, for moderate-to-complex tasks, individuals utilized more cortical resources, leading to the result of positive correlations between brain operation and cognitive ability (Gevins and Smith, 2000; Neubauer et al., 2004; Papousek and Schulter, 2004). According to Vickers and Williams (2017), “this view challenges the widespread assumption that if an athlete is able to move quickly, then his or her neural processes must also function as fast or even faster” (p. 6). The purpose of this study was to systematically review self-paced (SP) and externally paced (EP) skills sport-related NEH research incorporating sport-related and simple discrimination tasks along with functional neuroimaging or brain stimulation. The framing question for this review was: How does long-term specialized training change athlete's brain and improve efficiency? In this review, “long-term specialized training” is defined as a planned, structured and progressive development of sport-specific skill to achieve better performance and competitive longevity (Granacher and Borde, 2017).

## Method

### Search and Screening

**Figure 1:**
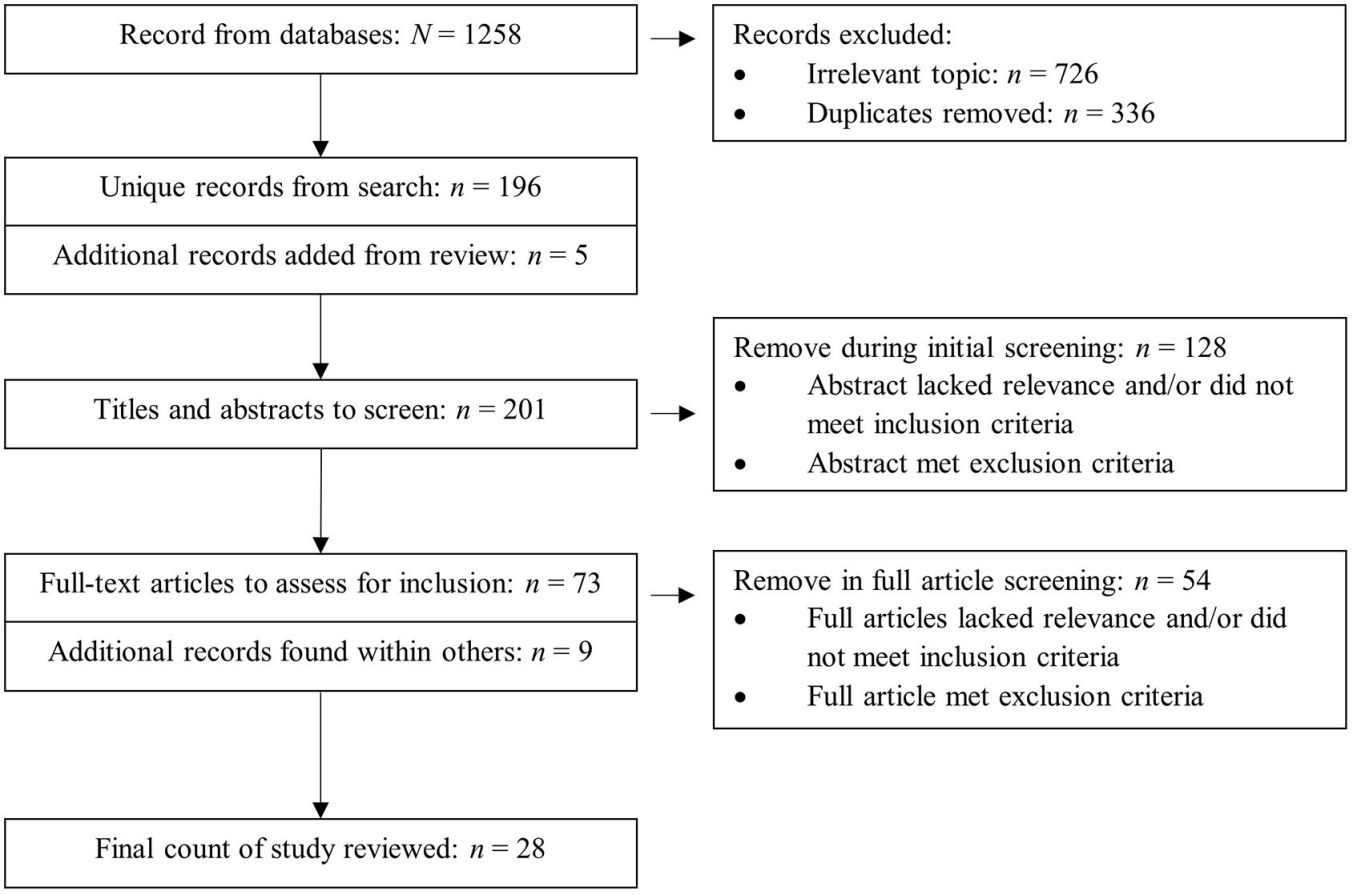
PRISMA flowchart of search strategy and screening.

The search strategy included the use of the following databases: PubMed, SPORTDiscus, PsycInfo, MEDLINE Complete, Education Resources Information Center (ERIC), Dimensions, and Google Scholar. Keyword combinations and MeSH terms employed in the search are listed in Table 1. Ten additional references were retrieved from the reference lists of the following papers in addition to the database search: Eslinger and Tranel (2005), Nishiguchi et al. (2015), Park et al. (2015), Di Fronso et al. (2016), Fargier et al. (2017), Gourgouvelis et al. (2017), Yamashiro et al. (2018), Hwang et al. (2019), Nakata (2020), and Parr et al. (2020). As for the additional references, Wei and Li (2017) and Wei and Li (2018), which were written in Chinese, were found within full-text articles being screened for inclusion. The four-phase PRISMA flow diagram (Figure 1) illustrates the search and screening process.

**Table 1:** Flowchart of search strategy.

**Concept**	**Keywords**
I. Outcome: Neural Efficiency	“Neural efficiency” or efficiency or plasticity or electrophysiology or “brain waves” or evoked potentials or visual evoked response or neurology or activation or motor-related cortical potentials or MRCPs or blood oxygenation level-dependent or BOLD or event-related potentials or ERPs or event-related desynchronization or ERD or event-related synchronization or ERS or motor cortex [MeSH Terms] or visual cortex [MeSH Terms] or neuronal plasticity [MeSH Terms] or motor skill [MeSH Terms] or cognition [MeSH Terms] or brain [MeSH Terms] or cerebral cortex [MeSH Terms] or functional connectivity [MeSH Terms] or cerebellum [MeSH Terms]
II. Participants: Children, Adolescents, and Adults	Youth or adolescents or young people or teen or young adult* or children or kids or adult* or middle aged or humans or male or female or healthy
III. Exposure: Expert and Novice	Athlet* or sport* or expert* or elite* or novice or non-athlet* or subject* or “expert-novice paradigm”
IV. Exposure: Exercise Types	Physical activity or exercise or fitness or physical exercise or sport* or self-paced or externally paced or open skill or closed skill or long-term or specific or training
V. Design and Neurophysiological Techniques	Experimental* or quasi experimental* or observational* or randomized control trial or cross sectional* or case-control*or functional neuroimaging [MeSH Terms] or computed tomography or CT or positron emission tomography or PET or electroencephalogram or EEG or magnetic resonance imaging or MRI or functional magnetic resonance imaging or fMRI or magnetoencephalography or MEG, near infrared spectroscopy or NIRS or transcranial magnetic stimulation or TMS

Bibliographic databases: PubMed, SPORTDiscus, APA PsycInfo, MEDLINE Complete, ERIC, and Dimensions; We used “OR” to separate keywords within each concept and “AND” to separate each concept; MeSH terms were used for PubMed search only; Google Scholar was used for cross validation for search results from multiple bibliographic databases. The latest search of the aforementioned databases was on June 18, 2021. Symbol * is a truncation operator (e.g., athlet* is a stem that would return matches with athlete, athletes, athletic, etc.).

Inclusion criteria were as follows. First, the study sample included participants under 65 without any cognitive and brain diseases. Second, at least part of the study sample consisted of “experts,” defined as subjects highly trained for specific skills (Grabner et al., 2006). That is, within the study sample, at least one group of participants were recognized as having expert domain-specific motor skills (e.g., superior skills as athletes in either individual or group sport, etc.). Both SP skills, which are initiated by the performer (e.g., golf putting, high jumping), and EP skills, in which the timing of performance of the skill is controlled by an outside influence/environment instead of the performer themselves (e.g., football passing, tennis rally), were included (Singer, 2000). Third, the study protocol included each of the following: (1) a resting state eyes-closed and eyes-open recording; (2) a visual stimulus consisting of a domain-specific motor action sequence (e.g., simple discrimination or multiple object tracking task, viewing videos or pictures of table tennis serves, rhythmic gymnastics, football actions, etc.) with a general or sport-specific challenge to simulate the stress of high-level competition; and (3) a decision task in which participants reacted to the visual stimulus (e.g., completing a scale to report mental stress, completing a cognitive assessment, evaluating motor performances, matching targets by pressing a number key to indicate the number of probe items, or executing motor movements such as karate punches). Fourth, at least one functional neuroimaging or stimulation technique was used in this research (e.g., PET, fMRI, fNIRS, or EEG) to assess brain regions activation during the tasks. Specifically, activation data including ERPs (amplitude and latency), BOLD signals of cerebral activation, and oxygenated hemoglobin (HbO), deoxygenated hemoglobin (HbR), and total hemoglobin (HbT) levels between experts and novices. Fifth and finally, the study was an original empirical study written in English or Chinese and published in last two decades (2001–2021). Consequently, studies were excluded if they did not apply expert-novice comparison or outcome excluded cognitive function, motor execution, cortex activation, and functional connectivity (FC), and neither neuroimaging nor stimulation were used meta-analyses or other systematic reviews were excluded.

### Data Extraction and Analysis

Several characteristics were examined for any of the studies that are part of this review: expert-novice samples, visual stimulus tasks, neuroimaging and stimulation techniques, and essential conclusions. The criterion for emphasizing these features was to incorporate standard observations, including brain structures involving, uncover new findings, and recognize methodological strengths and shortcomings, allowing recommendations for future scientific research.

On the basis of similarity and differences within the selected studies, we applied co-authorship and co-occurrence analysis to explore the collaborations among researchers and key concepts via VOSviewer (Version 1.6.16; Van Eck and Waltman, 2010). This procedure was designed to recognize the research collaborations and themes and trends in the field of neural behavior and neuroimaging. According to Van Eck and Waltman (2010), a full counting co-authorship network was constructed based on authors, organizations, and countries and a co-occurrence map was constructed in a three-step process: first, the co-occurrence matrix was used to construct a similarity matrix (see Supplementary Material); second, the VOS mapping technique was used to establish a map from the similarity matrix, which was weighted on total link strength; finally, the map was translated, rotated, and reflected.

## Results

As shown in Table 2, the frequencies of the sports, countries, and neuroimaging types represented across the 28 included studies with reference numbers to refer to the studies hereafter (numbered according to alphabetical order). The median publication year of these studies was 2014 (mean = 2013; *SD* = 5.43; range = 2001–2020). These studies (*N* = 829) had a mean total sample size of 29.61 (*SD* = 15.14; range = 7–81) and a mean expert group sample size of 15.37 (*SD* = 8.77; range = 1–42).

**Table 2:** Frequencies of expert sports, countries, and neurophysiological techniques in selected studies (*N* = 28).

	**Reference numbers**	**No. of studies**
Karate	2, 3, 5–8, 11	7
Table tennis	12, 24, 25, 27	4
Soccer	9, 14, 18	3
Basketball	22, 28	2
Archery	15, 21	2
Badminton	23, 27	2
Baseball	19, 26	2
Kendo	13, 16	2
Football	4	1
Tennis	27	1
Rhythmic Gymnastics	1	1
Gymnastics	16	1
Volleyball	28	1
Fencing	5	1
Swimming	18	1
Shooting	10	1
High Jump	20	1
Golf	17	1
Italy	1–3, 5–10	9
China	12, 22–25, 27, 28	7
Japan	13, 14, 16, 18, 19, 26	6
Korea	15, 21	2
United States	4, 17	2
Turkey	11	1
Sweden	20	1
EEG	1, 2, 5–11, 13, 14, 16, 19, 23–26	17
fRMI	3, 4, 11, 12, 15, 17, 18, 20, 22, 27, 28	11
fNIRS	21	1

*1 = Babiloni et al. (2009); 2 = Babiloni et al. (2010); 3 = Berti et al. (2019); 4= Costanzo et al. (2016); 5 = Del Percio et al. (2008); 6 = Del Percio et al. (2009b); 7 = Del Percio et al. (2010); 8 = Del Percio et al. (2011); 9 = Del Percio et al. (2019); 10 = Di Russo et al. (2005); 11 = Duru and Assem (2018); 12 = Guo et al. (2017); 13 = Hatta et al. (2009); 14 = Iwadate et al. (2005); 15 = Kim et al. (2014); 16 = Kita et al. (2001); 17 = Milton et al. (2007); 18 = Naito and Hirose (2014); 19 = Nakamoto and Mori (2008); 20 = Olsson et al. (2008); 21 = Park et al. (2020); 22 = Qiu et al. (2019); 23 = Wang and Tu (2017), 24 = Wei and Li (2017); 25 = (Wei and Li, 2018); 26 = Yamashiro et al. (2015); 27 = Yang et al. (2020); 28 = Zhang et al. (2019)*.

*fMRI, Functional magnetic resonance imaging; EEG, Electroencephalogram; fNIRS, Functional near-infrared spectroscopy*.

*Duru and Assem (2018) used both fMRI and EEG in their study; Berti et al. (2019) and Yang et al. (2020) applied rs-fMRI in their study*.

### Expert-Novice Samples

The expert-novice paradigm is commonly used in neurobehavioral research (Callan and Naito, 2014). However, the definition of expertise or experts are inconsistent across expertise studies (Baker et al., 2015). Given the taxonomy by Baker et al. (2015), novices are defined as individuals in early phrases of skill development with limited skill, and experts are eminent athletes reaching peak levels of skill. Although the standard of identifying experts in selected studies varies, the years of experience and belonging to athletic teams (e.g., national team) are two consistent criteria. Studies 4, 6, 14–16, 19, and 20 did not include years of experience data, but participants in expert groups were either experienced collegiate athletes (4, 14, 19) or national team members/top-ranking athletes in their countries (6, 15, 16, 20). The original experimental group terms use in selected studies are listed in Table 3. The competitive level of expert groups ranged from professional or elite athletes (1, 2, 6, 7, 10, 15, 18, 20, 21) to national level, regional, or collegiate athletes (3, 4, 5, 8, 9, 11, 12–14, 16, 17, 19, 22–27). Among studies, experts reported the number of years for their participation in the perspective sports, and the mean of these is 11.27 (*SD* = 4.96) years. A range of approaches to sampling was apparent across the 28 studies. Noteworthily, participants in study 15 included Olympic gold medallist archers, and study 18 included professional soccer player Neymar. These participants had 17.83 and 16 years of experience in their respective sports.

**Table 3:** Extraction table of the studies examining the neural efficiency hypothesis in experts-novices paradigm.

**References**	**EG Term used (mean experience yrs.)**	**Neurophysiological techniques**	**EG skill type**	**NEH supported? (compared to CG/Novice)**	**Controversies/non-significances**
Babiloni et al. (2009)	Elite (8+)	EEG	SP	Spatially selective cortical activation ↓ (low- and high-frequency alpha ERD was lower in amplitude in occipital and temporal areas and in dorsal pathways, at right hemisphere (visuo-spatial selective attention)	Both experts' hemispheres in the whole video's duration were involved for the best judgment instead of only right visuospatial hemisphere activated
Babiloni et al. (2010)	Elite/Amateur (12+; 2–5)	EEG	EP	Dorsal and mirror pathways (lower alpha ERD, elite < amateur < novice)	Low frequency alpha ERD/S in ventral pathway showed no difference (*p* > 0.10)
Berti et al. (2019)	Elite (14)	rs-fMRI	EP	Increase FC between the right superior parietal lobe, bilateral occipital poles, and auditory and motor-related areas (possibly driven by long-term specific training)	Increased positive correlation in occipital-parietal-temporal network with left hemisphere more prominently involved in experts
Costanzo et al. (2016)	Collegiate Athlete (n.r.)	fMRI	Mix	Prefrontal areas and insula demonstrated NE BOLD during exposed to unpleasant stimulus	Observed activation in the amygdala was not significant in EG and CG comparisons
Del Percio et al. (2008)	Elite/Elite (10+)	EEG	SP/EP	Supplementary motor and contralateral sensorimotor areas with ↓ RP and motor potentials	MP amplitude over ipsilateral sensorimotor area was higher in the karate than fencing elites; NE depends on side of the movement, hemisphere, and athlete's trait
Del Percio et al. (2009b)	Athlete/Athlete (n.r.)	EEG	SP/ EP	Left central, right central, middle parietal, and right parietal areas (↓ low-frequency alpha TRPD, *p* < 0.01); Right frontal, left central, right central, and middle parietal areas, *p <* 0.03 (↓ high-frequency alpha TRPD)	Alpha ERD for eyes-open referenced to -closed (upright bipodalic standing) was higher in amplitude in experts
Del Percio et al. (2010)	Elite Athlete (12+)	EEG	EP	Primary motor area, lateral and medial premotor areas, *ps* < 0.0005–0.005 (↓ high frequency alpha ERD was lower in amplitude in both preparation and execution of the right movements)	Unclear the reason of NEH was more represented in right (dominant) than left movements)
Del Percio et al. (2011)	Athlete (12+)	EEG	EP	Frontal (*p* < 0.00002), central (*p* < 0.008), right occipital (*p* < 0.02) areas (↓ low frequency alpha TRPD); frontal (*p* < 0.00009) and central (*p* < 0.01) areas (↓ high-frequency alpha TRPD)	Reduction of alpha power for eye-open to close condition (upright bipodalic standing) was greater in experts
Del Percio et al. (2019)	Player (12.7)	EEG	Mix	n.r.	A prominent and bilateral parietal alpha ERD was greater (*p* < 0.05) in experts (Low-frequency alpha sub-band: P4, *p* = 0.01; high-frequency alpha sub-band: C4, *p* = 0.01, P3, *p* = 0.04, P4, *p* = 0.002)
Di Russo et al. (2005)	Professional Athlete (6+)	EEG	EP	BP and NS′s components related to right finger movements had a later (*p* < 0.01) onset and reduced (BP, *p* < 0.005; NS′, *p* < 0.01) amplitude in the left SMA and PMA	No difference was found between expert and novice for MP and RAP
Duru and Assem (2018)	Elite (7+)	fMRI + EEG	EP	Frontal (alpha, *p* < 0.009; beta, *p* < 0.029), midline (alpha, *p* < 0.004; beta, *p* < 0.043), parietal occipital (alpha, *p* < 0.072; beta, *p* < 0.081), and RCT regions (alpha, *p* < 0.0001) ↓ ERD/S	n.r.
Guo et al. (2017)	Athlete (8+)	fMRI	Mix	Bilateral middle frontal gyrus; right middle orbitofrontal area, SMA, paracentral lobule, precuneus, angular gyrus; left supramarginal gyrus, inferior temporal gyrus; middle temporal gyrus, bilateral lingual gyrus and left cerebellum crus ↓	Precuneus showed ↑ under the sports related vs. unrelated stimulus condition in experts
Hatta et al. (2009)	Player (16.4)	EEG	EP	Shorter BP latencies for the non-dominant handgrip task	BP onset time for non-dominant handgrip task was earlier in control (*p* < 0.001); MP amplitudes in experts were significantly larger (*p* < 0.001) than novices
Iwadate et al. (2005)	Collegiate Athlete (n.r.)	EEG	Mix	P300 latency was significant shorter in lower-limb task (*p* < 0.05)	Increased P300 amplitudes (*p* < 0.001) and reduced latencies during the lower-limb task (*p* < 0.01); larger N140 amplitudes during both the upper- and lower-limb tasks (*p* < 0.01); no significant (*p* = 0.78) difference in the upper-limb task
Kim et al. (2014)	Elite (17.8), Expert (11.9)	fMRI	SP	Left superior and inferior frontal areas, ventral prefrontal cortex, right SMA, right primary somatosensory area, and left precuneus, both temporoparietal areas, the left PCC, the right BG, and left cerebellar nodule and tonsil ↓	Right SMA, MFC, the right and left temporoparietal area, and the declive and dentate of the right cerebellum ↑ in elite; ACC (similarity in activation levels between elites and novices, but not experts)
Kita et al. (2001)	Athlete/Athlete (n.r.)	EEG	SP/EP	MRCPs onset time were shorter (*p* < .01); amplitudes of BP were smaller preceding wrist extensions in the contralateral motor area (*p* < .01)	No significant difference in NS′ and MP amplitude between EG and CG
Milton et al. (2007)	Expert (n.r.)	fMRI	SP	BG (*p* < 0.02), LIMBIC (*p* < 0.0002) ↓	Cortical regions (SPL, LPMCd, OCC) ↑ in experts
Naito and Hirose (2014)	Professional (16+)/Elite (9+)	fMRI	SP/Mix	The size and intensity of medial-wall activity in foot M1 ↓ (*p* < 0.05) both in size and strength	The size and intensity of medial-wall activity was smaller in other participants besides Neymar
Nakamoto and Mori (2008)	Collegiate Athlete (7-12)	EEG	Mix	Shorter (*p* < 0.01) interval between stimulus and LRP onset (in Go trails)	Augmented P3 amplitude, spatial-BB (*p* < 0.01) and color (*p* < 0. 05) tasks in experts in frontal (Fz) in Nogo trials
Olsson et al. (2008)	Elite (n.r.)	fMRI	SP	Visual and parietal cortex (superior occipital lobe and inferior parietal cortex) ↓ in CG and non-imagery trained athletes (*p* < 0.05). Significant left lateralization (*p* < 0.05)	Bilateral pre-motor cortex, SMA and Cerebellum ↑ in experts, on the right side (*p* < 0.05)
Park et al. (2020)	Elite (n.r.)	fNIRS	SP	More stable pattern of variability in hemodynamic responses (HbO, HbR, and HbT) from prefrontal cortex	No group difference in overall average HbO, HbR, and HbT responses from PFC and DLPFC
Qiu et al. (2019)	Athletes (6.5)	fMRI	Mix	Left FEF, MTG, bilateral aIPS in the MOT task, and core part of DAN ↓; as attentional load increased, deactivation of left MTG differences become larger between EG and CG	Left temporal ↑ in experts
Wang and Tu (2017)	Collegiate Athlete (5+)	EEG	Mix	Lower amounts of attentional resources of irrespective information control (smaller CNV amplitude in the condition involving low uncertainty, *p* = 0.018)	Greater P3 amplitudes (*p* = 0.01) in experts
Wei and Li (2017)	Experts (10)	EEG	Mix	Occipital N1 (*p* < 0.01); Frontal, parietal, and central P3 (*p* < 0.01) lower amplitude; parietal-central area alpha ERD ↓ (*p* < 0.01)	Frontal and central N1 and N2 (higher amplitude, *p* < 0.01) in experts
Wei and Li (2018)	Experts (10)	EEG	Mix	Occipital-parietal visual and MNS cortices theta and alpha ERP ↓ (*p* < 0.05); right hemisphere task region (increased effective FC); left hemisphere and inter-hemispheric region (decreased inefficient FC)	Right occipital-temporal (*p* < 0.05) ↑ and right frontal-temporal cortices ↑ (*p* < 0.01)
Yamashiro et al. (2015)	Collegiate Athlete (9+)	EEG	Mix	The peak latency of inhibition of movements (Nogo-N2 were shorter, *p* < 0.05)	Frontal area Nogo-N2 (Larger amplitude, *p* < 0.05) in experts; negative correlation between Nogo-N2 (*r* = 0.50, *p* < 0.05) and Nogo-P3 (*r* = 0.53, *p* < 0.01) potentials and RT
Yang et al. (2020)	Collegiate Athlete (3+)	rs-fMRI	Mix	Left triangular part of the IFG, extending to the opercular part of the left IFG and middle frontal gyrus (↓ gFCD, *p* < 0.05); positive correlation between gFCD and RT (*r* = 0.46, *p* = 0.0021)	Left superior parietal lobule and the left MFG, lower FC in experts
Zhang et al. (2019)	Expert (9.8–10.7)	fMRI	Mix	Left putamen, inferior parietal lobule, SMA, postcentral gyrus, right insula ↓; better temporal congruence between motor executions and motor imagery (*p* < 0.001); effective in the representation and the interoception of the motor sequences in volleyball (*p* < 0.001) and basketball (*p* < 0.01) experts	Experts involved more efficient motor simulation and less neural effort in performing the integrated representation of their self-sport

*↓ = decreased activation; ↑ = increased activation; Mix = The study includes the type of sport appeared both self-paced as well as externally paced characteristics, such as tennis, serving is self-paced but rally is externally paced; SP/EP = The study includes both self-paced as well as externally paced sports in either expert or novice group*.

The most popular approach was to investigate one sample of expert athletes and one sample of participants who have never practiced in the sport before (i.e., non-athlete or novice group), which aligned with studies 1–17, 19–22, 24, 25, and 27. In study 5–7 and 9, age and gender were used as covariates in the subsequent statistical study to rule out the possibility that minor variations in age and gender impacted the final statistical results. Study 2, 5, 6, and 16 explicitly divided participants into three levels of experience (elite, amateur, and athletes in other sports). Similarly, study 2 had three groups (elite, amateur, and novice) consisting of 12 years, 2–5 years, and no experience playing karate, respectively. The novice group (2, 5, 7, 8) did not reach the competitive or amateur level of playing karate or other sports similar to karate (i.e., kung fu, etc.).

Study 23, 26, and 28 took a unique approach in which expert athletes and tasks from two separate sports were recruited, basketball and volleyball, baseball comparing to Track and Field and Swimming, as well as badminton and track and field, respectively. In this way, each group operated in both roles of experts and novices in different sports. Noteworthily, in study 24 and 25, both experts and novice participants (*n* = 39) were selected randomly, 19 national level competitors (Age = 20.2 ± 1.2) and 20 novice university students (Age = 20.8 ± 0.9), respectively. In studies 1–10, 12–17, 20–28 participants were right-handed and age and gender were equally balanced in expert and non-expert groups.

### Perceptual-Cognitive Tasks and Neuroimaging Technologies

In EEG (1, 2, 5–8, 11, 12 13, 14, 16, 19, 23–26), fMRI (3, 4, 11, 12, 15, 17, 18, 20, 22, 27, 28), and fNIRS (21) studies the subjects were comfortably seated in front of a computer monitor with a distance of 60–95 cm to the monitor. Subjects watched the domain-specific motor action sequence videos as the visual stimulus. Examples included simple discrimination tasks (24) or MOT task (22), table tennis serves (25), rhythmic gymnastics (1), and football actions (4). After each video stimulus, participants were required to make a decision in response to the visual stimulus. Examples included completing a scale to report mental stress, completing a cognitive assessment, evaluating motor performances, matching targets by pressing a number key (5–7, 9, 10, 12, 15, 17, 18, 22, 23, 27), moving a joystick to indicate the number of probe items (4), and executing motor movements such as karate punches. In order to collect behavior data, in some studies (e.g., 1, 2), subject's personal coach or similar level expertise completed the same evaluation. The coach's evaluation or other pre-established criteria was used as the gold standard for assessing action judgment in the current participants to analysis the behavioral data (e.g., judgment error). Moreover, tasks also involved decision-making (left vs. right, hit vs. miss, etc.). In fMRI studies (3, 4, 11, 12, 15, 17, 18, 20, 22, 27, 28), experimental procedures were similar to EEG studies in that participants were presented with visual stimuli showing sport event-related design videos (similar to the studies reviewed by Smith, 2016). Participants evaluated the performance related to the video and responded by pressing a button (14), directing a joystick (5) or a motor action when the imperative stimulus appeared (4). Across the selected studies, participants observed video clips of rhythmic gymnastics performance (1, 7), karate (5, 6), motor control in soccer (18), negative sense in football (4), table tennis tasks (12, 18, 27), fencing attacks (5), archer aims at the target (15), basketball feel throws (22), and badminton serves (23, 27). All studies had provided associated criteria with the task.

In this review, we found there to be three distinct advantages of using EEG (17 studies), compared to using fMRI (11 studies) or fNIRS (one study). First, “studies can be carried out in the live ‘*in situ*’ setting in sports,” such as rhythmic gymnastics, archery, table tennis and fencing (5, 12, 24, 25, 27), thereby allowing the measurement of neural activation as specific sport tasks are performed successfully or unsuccessfully” (Vickers and Williams, 2017, p. 15). Second, EEG studies (1, 2, 5–8 11, 12 13, 14, 16, 19, 23–26) provide “precise measurement of the temporal activation of neural networks as movements are prepared,” unlike fMRI (3, 4, 11, 12, 15, 17, 18, 20, 22, 27, 28), which lacks the temporal resolution to provide this information (Vickers and Williams, 2017, p. 15). Third, eye movement potentials “can be determined as EEG is recorded” (1, 2, 5, 6–9, 19, 23), “thereby providing insight into the spatial locations of gaze fixations and the duration of focus on critical cues” (Vickers and Williams, 2017, p. 15). Quiet eye also identifies “the critical phase of the movement when the QE must be focused to lead to successful vs. unsuccessful trials” (Vickers and Williams, 2017, p. 15; Mann et al., 2016). Meanwhile, EEG studies (23, 25) that determined theta activation levels in table tennis serve are reviewed, as well as studies (5–7) that have determined the EEG, EOG, and EMG concurrently. In sum, all studies applied at least one neuropsychology technology consisting of fNIRS, fMRI, or EEG (study 11 used both fMRI and EEG) to examine areas of neural activation during event-related stimuli controlled for baseline activation, except for study 18, in which investigators conducted one extra session to record participants' foot movements.

### Efficiency Paradox

**Figure 2:**
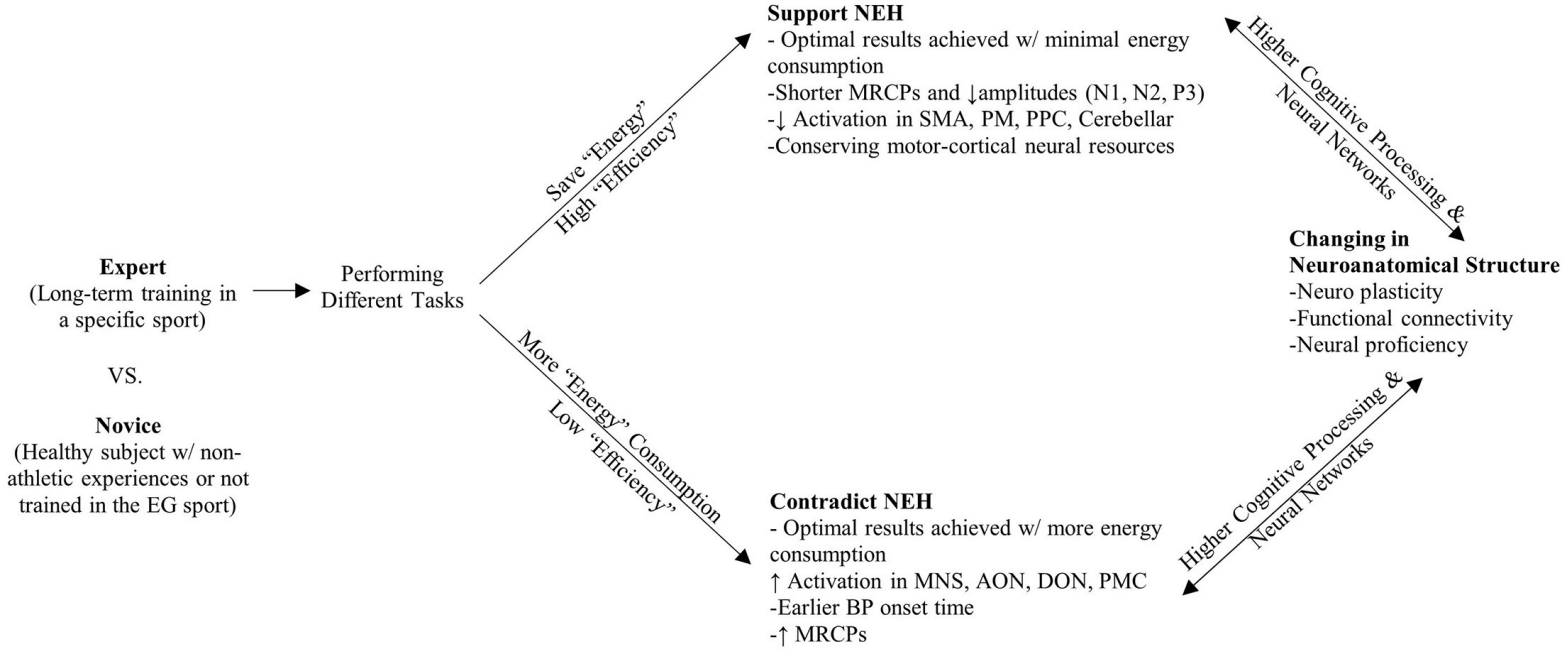
Schematic overview of neural efficiency in athletic brain.

We assume neural efficiency shown in athletes is considered as an integration of neuroanatomical structure changes (neural plasticity) and neural proficiency of higher cognitive processing and neural network through long-term training in specific sports. The accumulative evidence supported our assumption (Calvo-Merino et al., 2005; Rypma and Prabhakaran, 2009; Zamora-López et al., 2010; Paolicelli et al., 2011; Turella et al., 2013; Wolf et al., 2014; Nakata, 2020; Filho et al., 2021). In order to illuminate the mechanisms revealed in previous studies, a schematic overview of neural efficiency in the athletic brain is depicted in Figure 2. We extracted both supporting and conflicting evidence of the NEH and listed the key evidence in Table 3. Among all of the studies (except 9, 13), the revealed data can be theoretically interpreted in terms of neural efficiency in some degree. The summarized studies, in particular, typically report a negative association between brain activation and optimal task performance (1–8, 10–20, 22–28). This may indicate that experts use their brains more efficiently with less energy consumed (a smaller number of resources are allocated) than novices or non-athletes for performance of a task. To the extent that motor imagery may be seen as a preparation for execution, lateralization effect resulted in left hemispheric specialization in experts during execution of imagery based on established motor representations showed supportive evidence of the NEH (5, 7, 10, 17, 20), especially in right dominant sports (24, 25). Given the broad body of evidence supporting the NEH, we briefly summarize the findings that reported only partial support for the NEH; that is, only for certain categories (1–8, 10, 12–28), under specific conditions/tasks (6, 12, 19, 21, 24–28), for specific brain regions (1–8, 10–28), or even presented the opposite finding (1–10, 12–28). Moreover, the phenomenon of QE is introduced in the context of the efficiency paradox (Mann et al., 2016). But on the other hand, studies (1, 5–7, 9–11, 15, 22–24, 25–28) on complex visuospatial, visual search, motion observation, and cognitive tasks have shown that athletes' parietal, central, and other areas have higher cortical activation, inconsistent with the NEH. Possible reasons are as follows. First, the control group lacked specific experience and strategy, less task confidence and effort investment, less related neural cortex resource investment, and lower task-related brain activation. Second, as some authors have noted, it is possible that as activation in the Default Network (DN) increases, the athlete's control processing decreases (Calvo-Merino et al., 2005; Turella et al., 2013; Wolf et al., 2014). Third, long-term specific training may lead to the reorganization of cortical activation circuits and induce different cortical circuits. Moreover, considering the conflicting results, the phenomenon of QE was introduced in the context of the “efficiency paradox” (Mann et al., 2016).

Mann et al. (2016) identified an efficiency paradox that runs contrary to the NEH. The endorsement of a “longer is better” recommendation remains simplistic from both a scientific and intuitive standpoint, and the primary mechanisms correlated with this recommendation persist speculatively. However, extensive evidence emanating from previous studies shows that, paradoxically (i.e., the polar opposite), the QE control associated with superior motor skills is slower and of long duration. Even for tasks that are fast and ballistic, like table tennis serve (12, 24, 25, 27), the QE onset is early, on a specific location (4, 14, 22, 27, 26), and has a duration that is longer when identifying the opponent's movement than when reacting. Similarly, in soccer, badminton, and archery (15, 27), the QE tracking duration is longer on successful than on unsuccessful shots which the expert's cortex activation is greater than novice (2, 5, 9, 22, 26). Due to the limited capacity of cognitive capacity of human brains (3, 27), the athlete seems to find ways to navigate complex spatial information earlier and to maintain their focus under the most challenging of situations. Additionally, “at the highest competition level of sport, athletes are faced with immense levels of pressure, unpredictable playing conditions, and actions of opponents and officials that can be difficult to control” (Vickers and Williams, 2017, p. 9). Thus, the different perspectives related to the NEH need to be understood situationally. There are generally two categories of visual stimulus tasks in selected studies regarding the simple-moderate (e.g., discriminate color, shapes, or remain bi/mono-podalic upright standing) and moderate-complex (e.g., identify a backspin serving in table tennis from video clips or react to visual stimulus by executing motor movements) stimulus tasks. Compared to simple-moderate tasks, moderate-complex task involved more sports specific motor skills which possibly modulate high-level cognitive system resources allocating to task demands (Eng et al., 2005; Kliger and Yovel, 2020). In general, experts tended to perform better than novices in both types of visual stimuli tasks (simple-moderate: 6–8, 10–14, 16, 17, 19, 22, 24, 27; moderate-complex: 1–5, 15, 18, 20, 21, 23, 25, 26, 28) but the activation cortex areas and pathways are inconsistent (see Table 3). These results indicated that the judgment of observed sporting actions is linked to relatively lower levels of alpha ERD, which may be a sign of spatially selective cortical activation or neural efficiency (1–12, 14–28). Specifically, studies 14, 22, and 27 reported a further step in defining brain correlates of the NEH, aligned with Babiloni et al. (2010) which can be considered as a model of continuous plastic train-related adaptation in the aforementioned athletes, and studies (23, 28) concluded similarly when performing a task related to an individual's particular sport domain, competence in sports is correlated with proficient control of brain function during cognitive and motor preparation, as well as response execution (14, 21, 22). In fMRI studies (3, 27), specifically in the resting-state condition, there were reduced connections between brain regions (e.g., left IFG and MFG) and remaining brain voxels in experts. This aligns with the NEH because the reduced connections (i.e., conservation of resources), might reflect improved global efficiency in the athlete's brain. The supporting and contradictory evidence for the NEH is discussed further in the Discussion section.

### Results From Co-authorship and Co-occurrence Analysis

**Figure 3:**
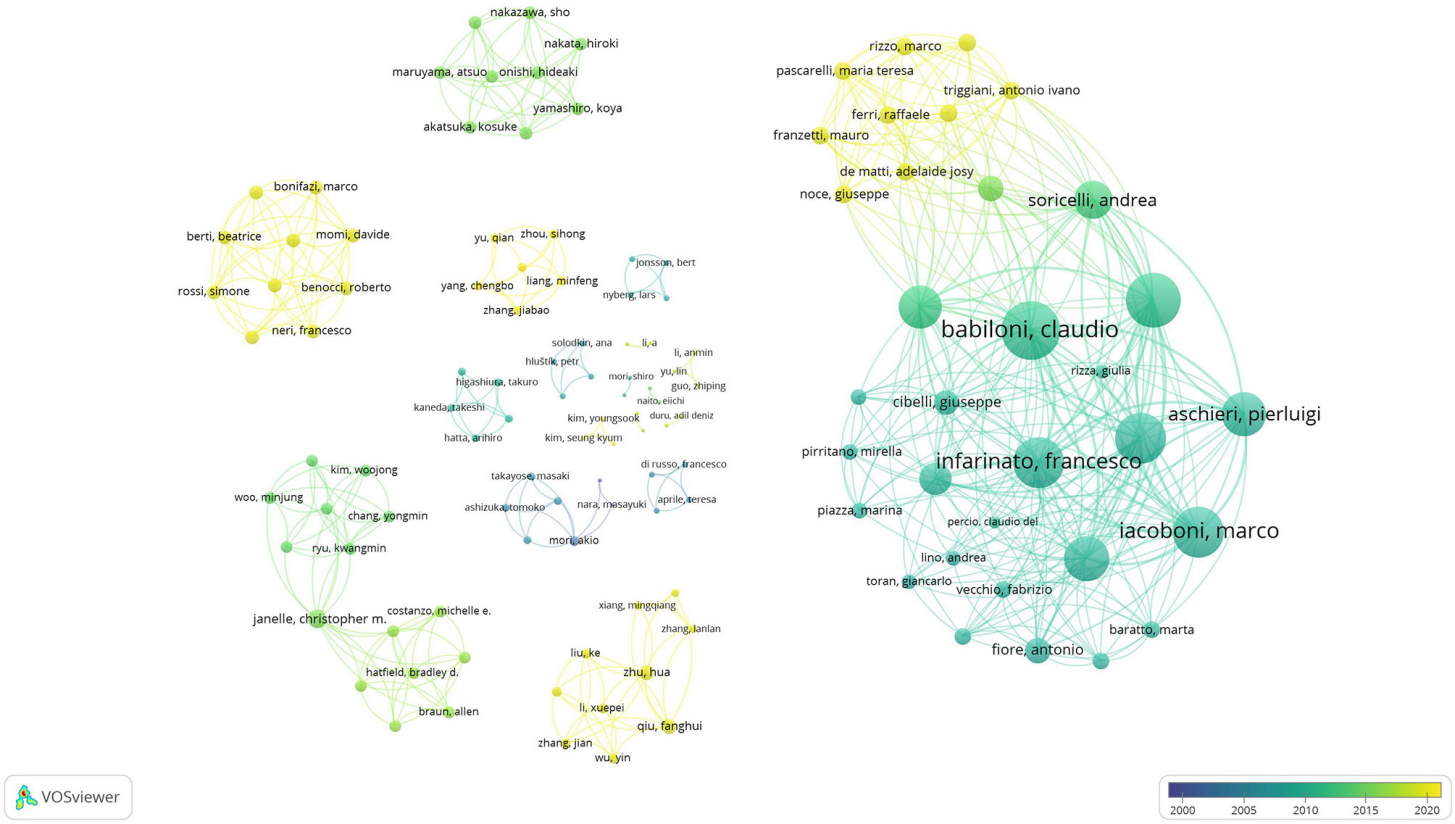
Co-authorship map.

**Figure 4:**
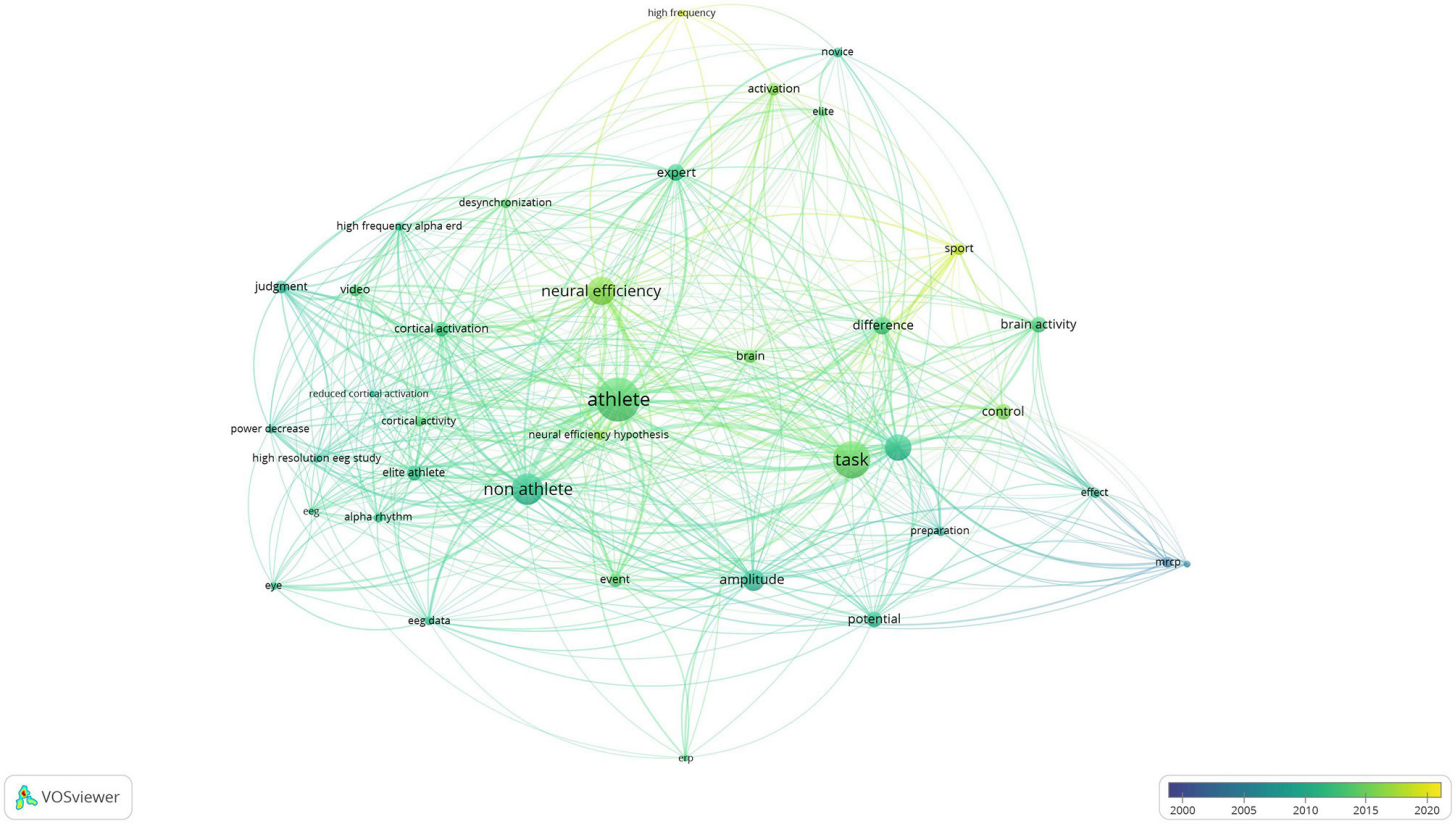
Co-occurrence map and key terms of selected studies.

The results of co-authorship and co-occurrence analysis were demonstrated regarding to two visualization maps (Figures 3, 4). In both maps, items (authorships and co-occurrence terms) are represented by their labels and circles. The size of the label and circle of an item is determined by the weight of the item (Van Eck and Waltman, 2010). Co-authorships were weighted on total link strength (items = 123, clusters = 19, links = 495, total link strength = 664, and Maximum iterations = 1,000). The demonstration of authorship clusters was verified across the studies. Study 12, 22, and 28 included a similar sample size, findings, and authors working collaboratively in the field. Authors from studies 1, 6, 24, and 25 shared similarities in sample size, imaging method, and task protocol. Authors from studies 5, 9 and 25 are decentralized because those studies concluded contradictory results (as discussed in the previous section). Lastly, clustering may be due to similarities in the imaging method and/or sample size, and/or the close collaboration among different research teams in different countries. By nations, co-authorships reflected that authors from Italy were most active with consistent and recent publications, followed by scholars from Japan, China, Korea, United States, Turkey, and Sweden, respectively (see Figure 3). The co-occurrence map was weighted on total link strength (items = 38, clusters = 4, links = 485, total link strength = 6195, and Maximum iterations = 1000). The transition of overlay from dark to light demonstrated the trends of published research from 2001 to 2020. The keyword and main results from the selected studies were categorized into clusters which demonstrated the core focus on athletes, non-athletes, neural efficiency, and tasks (see Figure 4). The results of co-authorship and co-occurrence analysis provided complimentary bibliography evidence that the expert-novice paradigm and NEH were popularized in most recent research associated with worldwide collaborations.

## Discussion

The studies included in this review had a number of strengths. First, the sample sizes of the median overall sample sizes and expert group sample sizes were 30 and 15, respectively, which is greater than is often found in functional brain imaging studies (Szucs and Ioannidis, 2020). Studies included sample sizes to achieve adequate statistical power (except for study 12; *N* = 7) and ensure balance between the sample sizes of expert and novice study groups. Second, the selected studies consistently took measures to account for many possible between-group confounding variables, such as age, vision, dominant hand, and psychological or neurological conditions. Studies 5, 6, 9, 27, and 28 were particularly strong in reporting possible confounding variables and the steps taken to control for them. Third, all studies also implemented within-participant controls by randomly presenting stimuli and applying multiple trials of each experimental condition.

There were also several limitations. First, in one study, the control group was used to compare cognitive performance while the control subjects did not perform any kinematic task (3). The basic movements of the karate discipline, however, restrict the FC analysis of athletes, and subjects in the control group cannot replicate these gestures (3). It is not reliable to conclude that “differences in FC results are specific for karate athletes, as athletes of other martial arts have not been tested” (Berti et al., 2019, p. 11). Second, the evidence found in the selected studies indicates that expert advantages in perceptual-cognitive tasks mainly occur in sport-specific tasks rather general tasks (1, 3 12, 19, 22, 24, 25). Therefore, it is still unclear whether a generic perceptual-cognitive training intervention can be effective to enhance sport-specific skills in athletes (Fleddermann et al., 2019). However, contrary to the NEH, the research on motion observation and representation tasks in dance and basketball shows that compared with novices, the mirror neuron system (MNS) of expert motion understanding is more activated or showing no difference (1, 2), which may be related to the moderating effect of task difficulty, DN, and confidence (Calvo-Merino et al., 2005; Turella et al., 2013; Wolf et al., 2014). Finally, it should be noted that all selected studies were cross-sectional studies (except study 20, consisting of a 6-week intervention without randomization) based on a comparison among two/three groups of participants. Accordingly, the investigators could not rule out that some differences may have already existed before practicing sports. Thus, more longitudinal studies are needed. In addition, it should be noted that many of the studies included in this review did not report effect sizes or enough information to enable readers to compute effect sizes.

The evidence presented above suggests that experts are more successful than novices when reacting to an upcoming event while recruiting less attentional resources and devoting more attention to subsequent goal analysis in more unexpected situations. This is in keeping with studies 12, 23, 24, 25, and 27, which asserted that players of racket sports demonstrate more attentional flexibility than novices. Despite the evidence that athletes have greater neuro-cognitive processing than novices, findings from study 7 might not be fully explained by the NEH. With respect to the NEH, experts have been shown to have lower cortical resource expenditure (i.e., more automaticity) than novices on several occasions. In study 23, despite evidence that badminton players process the cue more automatically than athletic controls, they seem to put more effort into goal processing. The neural proficiency hypothesis (NPH), a broader term, has been proposed to explore the connection between neural activation and superior performance (Bertollo et al., 2016). According to the NPH, an athlete's effort to sustain a high level of performance by moving proficiently between optimal-automatic and optimal-controlled performance states can modulate brain activity (28), implying complex shifts in the implementation of different strategies to maintain optimal performance (Bertollo et al., 2016). In this way, efficient and effortful processing during performance, the degree of effort as well as the cognitive demands of the task's control systems, can be modulated. Compared to the novices, the neural efficiency in experts (12) not only decreased in activation of the occipital-parietal visual cortex and mirror system cortex, but also increased effective FC and decreased inefficient FC (25, 27) in the specific brain region (Cook et al., 2014). In the meantime, athletes can economize attentional resources when processing sport-specific cues in order to respond accurately and quickly. Recently documented meta-analytic EEG evidence in SP sports has suggested that the NEH, transient hypofrontality, and neural proficiency are complementary to each other in optimal motor performance (Filho et al., 2021). Although Filho et al. reported a non-significant increase in alpha and decrease in theta activity in expert-novice paradigm, they argued that theoretically the ERP results are congruent with the NEH (Grabner et al., 2006; Dunst et al., 2014). Compared to the evidence from EP and mixed skill sports, the ERP and BOLD results in studies (11, 12, 22, 25) align with SP sports, and studies (2–4, 13, 14, 19, 23, 24, 26–28) revealed both supportive and contradictory outcomes with SP sports and the NEH. Therefore, the NEH is a dynamic and situational concept that depends on several agents including movement characteristics (e.g., side of the movement), hemisphere, and athletes' traits.

More generally, neural efficiency is analogous to driving the same route to/from work for several years. You become progressively familiar with the route, enabling you to drive faster and with lower gas consumption compared to your first time driving on these roads. However, this efficiency is situational, depending on other contextual factors such as the traffic, weather, and other states that may fluctuate from one day to the next (e.g., your mood, alertness, etc.). Therefore, neuroplasticity is ever-present in human life, and its characteristics are individual- and situation-dependent (Gourgouvelis et al., 2017; Berti et al., 2019).

## Summary and Future Directions

In this study, selection, information, and analysis meta-biases were controlled rigorously by following the pre-established systematic review methods (Page et al., 2021). Studies on the NEH of intelligence (Haier et al., 1988; Neubauer et al., 2002) have found that expert individuals have lower brain activity when performing cognitive tasks because they tend to activate only the brain areas that are needed to complete the task. This has been explained as resulting from a genetic predisposition combined with extensive training (Callan and Naito, 2014). We demonstrated that several previous studies supported this theory, although subsequent research uncovered conflicting data, a paradox, or found pathways that moderate the perceptual-cognitive brain activation relationship (Alves et al., 2013). In this way, neural efficiency may indeed act as a factor for expert-novice brain region activation distinction as the various sports involved in 28 studies. Moreover, neural performance appears to be related to the amount and quality of training received. We examined the neuroscientific evidence from those studies, which revealed that long-term specific training might improve athletes' top-down processing pathway connectivity (Oliver et al., 2020). It is beneficial to the effect of unconscious resources in the frontal region on motor processing in the early stage, which improves the efficiency in fast-task response performance. This change could save limited attention resources for succeeding activation in motor task processing. More specifically, the visual N1 component induced by frontal and central regions were associated with the early reaction preparation processing (Vogel and Luck, 2000). The higher amplitude of N1 provoked by sports in the parietal-central area indicated that athletes consume more attention resources in the early stage of behavioral response, which may be related to the long-term specialized training. In one study (17), athletes can respond more quickly through early attention processing. The amplitude of N1 induced by athletes' frontal region was higher than that of the control group. Accordingly, experts have the capacity to conduct fast and accurate movement to satisfy the need for rapid action response during performance, saving attention resources for late brain executive processing, and promoting the cerebral cortex neural efficiency.

From integrating the aforementioned evidence, we concluded that the neural efficiency phenomenon is most commonly observed for frontal brain areas when athletes are confronted with spatial and perceptual-cognitive tasks of low to moderate task complexity (Neubauer and Fink, 2009; Alves et al., 2013). Compared with the initial training, the activation of subjects' frontal-parietal cortex gradually decreased with the proficiency of the skills, which theoretically supported the NEH (Haier et al., 1992; Grabner et al., 2006; Bueichekú et al., 2016). However, in highly complex tasks, both experts and novices seem to be able to stimulate more cortical resources, thus from the perspective of proficiency in brain function, there is a positive correlation between brain use and cognitive ability (Neubauer and Fink, 2009). Therefore, the inconsistencies in selected studies suggests that there may be conditional limits on the neural efficiency of the cerebral cortex in athletes.

According to the current evidence derived from selected studies, the degree of effort, as well as the cognitive demands of the control systems involved in the task, will influence individuals' efficient and effortful processing during performance (4, 6–8, 10, 15, 17–19, 24, 28). Athletes could benefit from transcranial direct current stimulation of targeted brain areas to improve learning through enhanced long-term potentiation while performing the task (Coffman et al., 2014; Flöel, 2014; Prichard et al., 2014). However, it is possible that this reversal of brain activation-professional training relationship is caused by athletes' psychomotor decision to motivate more commitment as compared to the novices, and the novices actually have insufficient training that the task surpasses their neural efficacy (9, 10, 22). In this way, as a result of recent neuromodulation findings and neuroimage methods, there are four potential future directions that may assist to clarify the detailed mechanism of neural efficiency: neuroimage technologies, study design, interdisciplinary research, and heterogenous populations.

Neuroimaging technologies are mutually complementary. Synchronization of high frequency rhythmic waves (i.e., gamma), in the occipital cortex of the subjects indicated that the task induced the occipital cortex. Previous studies (23, 25) have confirmed that high-frequency rhythmic wave oscillation is closely related to cognitive processing. Further analysis of gamma and alpha rhythms is considered in the later stage oscillation characteristics will be possible to better reveal the mechanism of athletes' brain neural efficiency. Meanwhile, the synchronization of neural clusters in the brain regions reflects the information processing characteristics of the brain neural network. Therefore, future studies should consider the degree of connection between the activation of brain regions and the function of brain regions and reveal the neural processing characteristics of the athletes' brains and the physiological mechanism of the neural efficiency of motor mobilization from the perspective of the whole brain network. In addition, the lack of spatial resolution of EEG also restricts the accurate localization of oscillating signal elicitors. Therefore, as Study 11 conducted, it is necessary to consider the combination of EEG and fMRI technologies to further explore the neural efficiency of athletes' brains.

Further, more rigorous designs are encouraged in future NEH experiments. Several studies (e.g., 22, 24) used simple-moderate recognition tasks that were familiar to all subjects, ensuring the ecological validity of the study and inducing the same cortical pathway to improve the comparability of cortical neural efficiency among the subjects. However, using simple-moderate general tasks cannot guarantee the specificity of special task processing. Studies (e.g., 18, 23, 25) used moderate-complex specific motor tasks could not eliminate the influence of knowledge factors such as action recognition strategy on cortex activation and functional connection (Poldrack, 2015). Therefore, it is necessary to optimize the design of experimental tasks to ensure that the same cortical circuits are induced while avoiding the interference effect of experiential knowledge on the differentiation of neural cortex functions. Moreover, the neural efficiency of an athlete's brain is a reflection of the microcosmic functional mechanism of cerebral cortex neurons, which non-traumatic neuroimaging studies cannot comprehensively investigate (Kandel et al., 2000). Therefore, interdisciplinary research is promising as a complementary approach to further explore the NEH at the molecular level, such as using animal models.

Lastly, broader populations and tasks will likely reveal more complementary evidence. According to Wang et al. (2013), tennis players have better inhibitory control than swimmers and novices, implying that training in open skill sports might promote fundamental cognitive control. While the NEH has recently been applied to a number of moment-related tasks, it is unknown whether neural efficiency exists in the broader population and different type of sports (e.g., long term specific training in individual and team sports, or adults and under 18 population). Moreover, we notice relative research revealed substantial EEG and MRI data which is encouraging but not convenient for researchers to compare outcomes parallelly. In this way, although the heterogeneous outcomes might be one of the barriers, adding more meta-analysis to the field is encouraging because a single summary estimate is more rigorous than systematic reviews and offers quantitative evidence of the long-term training effect on athletes' brains. Of note, in the future, pronounced evidence of the NEH also might be beneficial to patients with brain damage or related disease (e.g., Alzheimer's, Autism, Attention Deficit Hyperactivity Disorder) in the field of neurorehabilitation. Appropriate dose of exercise intensity, duration, and frequency may be useful in efforts to improve FC and reduce irrelevant information processing in a long-term perspective. In conclusion, this study has provided a comprehensive review of studies of neural efficiency distinguishing expert and novice athletes, raising several issues and directions for future research.

## Data Availability Statement

The original contributions presented in the study are included in the article/Supplementary Material, further inquiries can be directed to the corresponding author/s.

## Author Contributions

LL and DS contributed to the conception and design of the study. LL wrote the first draft of the manuscript and critically revising the draft for important intellectual content. DS contributed to revising and approving the final version of the manuscript. All authors contributed to the article and approved the submitted version.

## Conflict of Interest

The authors declare that the research was conducted in the absence of any commercial or financial relationships that could be construed as a potential conflict of interest.

## Publisher's Note

All claims expressed in this article are solely those of the authors and do not necessarily represent those of their affiliated organizations, or those of the publisher, the editors and the reviewers. Any product that may be evaluated in this article, or claim that may be made by its manufacturer, is not guaranteed or endorsed by the publisher.

## Abbreviations

ACC: anterior cingulate cortex
aIPS: Anterior intraparietal sulcus
BG: Basal ganglia
BOLD: blood-oxygen level-dependent
BP: Bereitschaftspotential
CNV: contingent negative variation
DAN: Dorsal attention network
DLPFC: dorsolateral prefrontal cortex
EG: Experimental Group
EMG: electromyogram
EP: Externally-paced skill
ERD: Event-related desynchronization
ERS: Event-related synchronization
FC: Functional connectivity
FEF: Front eye field
gFCD: Global functional connectivity density
IFG: Inferior frontal gyrus
LIMBIC: Limbic area
LPMCd: Lateral premotor cortex dorsal
LRP: Lateralized readiness potential
MD: mean difference
MP: motor potentials
M1: Primary motor cortex
MFC: Middle frontal cortex
MFG: Middle frontal gyrus
MNS: mirror neuron system
MP: Motor potential
MPCRs: Movement-related cortical potentials
MTG: Middle temporal gyrus
n.r.: Not reported or not readily usable/applicable for this type of summary table
NE: Neural efficiency
NEH: Neural efficiency hypothesis
NS: negative slope
OCC: Occipital
PMA: Premotor area
RAP: Reafferent positivity
RCT: right centrotemporal regions
RCTs: Random Control Trials
RP: Readiness potential
rs-fMRI: Resting state functional magnetic resonance imaging
RT: Reaction time
SMA: Supplementary motor area
Spatial-BB: spatial condition with baseball batting-specific S–R mapping
SP: Self-paced skill
SPL: Superior parietal lobule
TRPD/TRPI: Task-related power decreases/increases
yrs.: years.
