# Corrigendum: Neural Efficiency in Athletes: A Systematic Review

**DOI:** 10.3389/fnbeh.2022.841772

**Published:** 2022-02-11

**Authors:** Longxi Li, Daniel M. Smith

**Affiliations:** Department of Physical Education and Health Education, Springfield College, Springfield, MA, United States

**Keywords:** neural efficiency, athletes, sports, neuroimaging, neuroscience

In the original article Vickers and Williams (2017) was not cited in the article. The citation has now been inserted in *Introduction, Paragraph 1, 3, and 4; Results, Perceptual-Cognitive Tasks and Neuroimaging Technologies, Paragraph 2; Results, Efficiency Paradox, Paragraph 2* and should read:

“As increasing levels of expertise are attained, there are measurable changes in neural activation” (Vickers and Williams, 2017, p. 5). Historically, the neural efficiency hypothesis (NEH) “was first proposed by Haier et al. (1988), who adopted positron emission tomography (PET) to determine the relationship between task performance and level of neural activation during the performance of intelligence tests” (Vickers and Williams, 2017, p. 5). Haier et al. found “an inverse relationship between brain glucose metabolism levels and the score obtained on the intelligence test” (Vickers and Williams, 2017, p. 5). Participants who “had high intelligence scores consumed less energy than those with lower scores and performed more quickly, leading the authors to suggest that superior intelligence was due to neural circuits that performed at faster speeds and with greater efficiency” (Vickers and Williams, 2017, p. 5). In general, neural efficiency consists of better performance during the repetition of a task (Babiloni et al., 2009), lower energy consumption in completing same performance (Zhang et al., 2019), and relatively less pronounced alpha ERD as a commonly used index of neural efficiency or spatially selective cortical activation (Del Percio et al., 2008; Babiloni et al., 2010). Higher neural efficiency is characterized by a bidirectional reduction phenomenon encompassing both reduced activation of areas associated with task execution and reduced deactivation of regions associated with irrelevant information processing (Qiu et al., 2019).
…
In this way, investigators in the domain of motor learning suggested that “skilled performance was defined by high levels of automaticity, minimum energy expenditure, and reduced movement times” (Schmidt and Lee, 2014; Vickers and Williams, 2017, p. 5). According to Vickers and Williams (2017), “these documented changes have led to a general ‘faster-is-better’ approach in terms of defining optimal motor behavior, brain function, and assumptions about how athletes should be trained” (p. 5). For instance, athletes are often instructed to “shift their gaze rapidly and accelerate their thought processes and movements to the point of reducing the level of conscious control of what they are doing” (Shepherd, 2015; Vickers and Williams, 2017, p. 5). However, reducing conscious control did not lead directly to the non-conscious processes. Although robust evidence was found through non-conscious contributions to action control were strong, the non-conscious and conscious action control were still not fully understood (Shepherd, 2015). Moreover, this runs counter to the literature on quiet eye (QE), which calls for the performer to maintain their visual focus and concentration on a specific location during a critical final phase of movement (Vickers, 2016). Formally, QE is defined as “the final fixation or tracking gaze that is located on a specific location or object in the task environment within 3° of visual angle (or less) for a minimum of 100 ms” (Vickers, 2016, p. 119). In a comprehensive review of intelligence and the NEH, “Neubauer and Fink (2009) reported 29 studies in support of the hypothesis, while 18 provided mixed support and nine had contradictory results” (Vickers and Williams, 2017, p. 5). According to Neubauer and Fink (2009), a possible reason for the contradictory results is the variability in task difficulty across the studies they reviewed. That is, some studies incorporated tasks that may not have been demanding enough to find support for the NEH.
…
In addition, Neubauer and Fink (2009) concluded that the neural efficiency was mostly observed for low-to-moderately difficult tasks and in the frontal lobe of the brain. However, for moderate-to-complex tasks, individuals utilized more cortical resources, leading to the result of positive correlations between brain operation and cognitive ability (Gevins and Smith, 2000; Neubauer et al., 2004; Papousek and Schulter, 2004). According to Vickers and Williams (2017), “this view challenges the widespread assumption that if an athlete is able to move quickly, then his or her neural processes must also function as fast or even faster” (p. 6). The purpose of this study was to systematically review self-paced (SP) and externally paced (EP) skills sport-related NEH research incorporating sport-related and simple discrimination tasks along with functional neuroimaging or brain stimulation. The framing question for this review was: How does long-term specialized training change athlete's brain and improve efficiency? In this review, “long-term specialized training” is defined as a planned, structured and progressive development of sport-specific skill to achieve better performance and competitive longevity (Granacher and Borde, 2017).
…
In this review, we found there to be three distinct advantages of using EEG (17 studies), compared to using fMRI (11 studies) or fNIRS (one study). First, “studies can be carried out in the live ‘*in situ*’ setting in sports,” such as rhythmic gymnastics, archery, table tennis and fencing (5, 12, 24, 25, 27), “thereby allowing the measurement of neural activation as specific sport tasks are performed successfully or unsuccessfully” (Vickers and Williams, 2017, p. 15). Second, EEG studies (1, 2, 5–8 11, 12 13, 14, 16, 19, 23–26) provide “precise measurement of the temporal activation of neural networks as movements are prepared,” unlike fMRI (3, 4, 11, 12, 15, 17, 18, 20, 22, 27, 28), which lacks the temporal resolution to provide this information (Vickers and Williams, 2017, p. 15). Third, eye movement potentials “can be determined as EEG is recorded” (1, 2, 5, 6–9, 19, 23), “thereby providing insight into the spatial locations of gaze fixations and the duration of focus on critical cues” (Vickers and Williams, 2017, p. 15). Quiet eye also identifies “the critical phase of the movement when the QE must be focused to lead to successful vs. unsuccessful trials” (Vickers and Williams, 2017, p. 15; Mann et al., 2016). Meanwhile, EEG studies (23, 25) that determined theta activation levels in table tennis serve are reviewed, as well as studies (5–7) that have determined the EEG, EOG, and EMG concurrently. In sum, all studies applied at least one neuropsychology technology consisting of fNIRS, fMRI, or EEG (study 11 used both fMRI and EEG) to examine areas of neural activation during event-related stimuli controlled for baseline activation, except for study 18, in which investigators conducted one extra session to record participants' foot movements.
…
Mann et al. (2016) identified an efficiency paradox that runs contrary to the NEH. The endorsement of a “longer is better” recommendation remains simplistic from both a scientific and intuitive standpoint, and the primary mechanisms correlated with this recommendation persist speculatively. However, extensive evidence emanating from previous studies shows that, paradoxically (i.e., the polar opposite), the QE control associated with superior motor skills is slower and of long duration. Even for tasks that are fast and ballistic, like table tennis serve (12, 24, 25, 27), the QE onset is early, on a specific location (4, 14, 22, 27, 26), and has a duration that is longer when identifying the opponent's movement than when reacting. Similarly, in soccer, badminton, and archery (15, 27), the QE tracking duration is longer on successful than on unsuccessful shots which the expert's cortex activation is greater than novice (2, 5, 9, 22, 26). Due to the limited capacity of cognitive capacity of human brains (3, 27), the athlete seems to find ways to navigate complex spatial information earlier and to maintain their focus under the most challenging of situations. Additionally, “at the highest competition level of sport, athletes are faced with immense levels of pressure, unpredictable playing conditions, and actions of opponents and officials that can be difficult to control” (Vickers and Williams, 2017, p. 9). Thus, the different perspectives related to the NEH need to be understood situationally. There are generally two categories of visual stimulus tasks in selected studies regarding the simple-moderate (e.g., discriminate color, shapes, or remain bi/mono-podalic upright standing) and moderate-complex (e.g., identify a backspin serving in table tennis from video clips or react to visual stimulus by executing motor movements) stimulus tasks. Compared to simple-moderate tasks, moderate-complex task involved more sports specific motor skills which possibly modulate high-level cognitive system resources allocating to task demands (Eng et al., 2005; Kliger and Yovel, 2020). In general, experts tended to perform better than novices in both types of visual stimuli tasks (simple-moderate: 6–8, 10–14, 16, 17, 19, 22, 24, 27; moderate-complex: 1–5, 15, 18, 20, 21, 23, 25, 26, 28) but the activation cortex areas and pathways are inconsistent (see Table 3). These results indicated that the judgment of observed sporting actions is linked to relatively lower levels of alpha ERD, which may be a sign of spatially selective cortical activation or neural efficiency (1–12, 14–28). Specifically, studies 14, 22, and 27 reported a further step in defining brain correlates of the NEH, aligned with Babiloni et al. (2010) which can be considered as a model of continuous plastic train-related adaptation in the aforementioned athletes, and studies (23, 28) concluded similarly when performing a task related to an individual's particular sport domain, competence in sports is correlated with proficient control of brain function during cognitive and motor preparation, as well as response execution (14, 21, 22). In fMRI studies (3, 27), specifically in the resting-state condition, there were reduced connections between brain regions (e.g., left IFG and MFG) and remaining brain voxels in experts. This aligns with the NEH because the reduced connections (i.e., conservation of resources), might reflect improved global efficiency in the athlete's brain. The supporting and contradictory evidence for the NEH is discussed further in the Discussion section.

The authors apologize for this error and state that this does not change the scientific conclusions of the article in any way. The original article has been updated.

## Publisher's Note

All claims expressed in this article are solely those of the authors and do not necessarily represent those of their affiliated organizations, or those of the publisher, the editors and the reviewers. Any product that may be evaluated in this article, or claim that may be made by its manufacturer, is not guaranteed or endorsed by the publisher.
