# Supplementary material for: Neural Efficiency in Athletes: A Systematic Review
Source: Front Behav Neurosci. 2021 Aug 5;15:698555. doi: 10.3389/fnbeh.2021.698555 (PMC8374331; doi:10.3389/fnbeh.2021.698555)
Supplement: Supplementary file 1 [file Data_Sheet_1.pdf]

Name	Study#	References
action	11	40
action anticipation task	1	1
action comprehension	1	1
action judgment	2	4
action observation	1	1
action planning	2	2
actions share	1	1
attack actions	1	1
certain action	1	1
conscious actions	1	1
defense actions	1	1
former action	1	1
goal-directed action	1	1
investigating action observation	1	1
judging sport actions	1	1
Judgment of observed karate actions	1	1
karate actions	2	2
motor action	1	2
nonpracticed actions	1	1
observed actions	1	2
observed sporting actions	2	4
of observed sporting actions	1	2
these actions	1	1
trained action	1	1
ongoing actions	1	1
paradigmatic football action	1	2
sporting actions	1	1
sport-related action anticipation	1	1
whole action	1	1
activation	14	42
activation cluster	1	1
bilateral activation	1	1
brain activation	3	3
brain activation pattern	1	1
circumscribed activation	1	1
cortical activation	4	5
cortical frontoparietal activations	1	2
decreased activation	1	1
efficient activation	1	1
frontoparietal activation	2	2
fronto-parietal activation	4	4
insula activation	1	1
intensive activation	2	2
investigating brain activation	1	2

medialwall activation	1	1
neural activation patterns	1	1
ofcortical activation	1	1
precuneus activation	1	1
preferential activation	1	1
reduced brain activation	1	1
regional activation	1	1
revealed activation	1	1
selective activation	1	1
showed activation	1	1
specific brain activation	1	1
task-relatedcortical activation	1	1
undifferentiated activation	1	1
unspecific muscle activations	2	2
activity	15	57
brain activity	6	9
brain electromagnetic activity	1	1
broad activity	1	1
concurrent activity	1	1
cortical activity	5	9
dorsal stream activity	1	1
eeg activity	1	1
event-related potentials activity	1	2
extensive executive control activities	1	1
global brain activity	1	1
hemodynamic cerebral activity	1	1
medial-wall activity	1	2
metabolic activity	2	2
mirror neuron activity	1	1
muscle activity	1	1
network activity	1	1
neural activity	7	12
neural activity changes	1	2
ofcortical activity	1	1
post-response activity	1	1
previous brain activity studies	1	2
scanned brain activity	1	1
sport activity	1	2
whole-brain activity	1	1
alpha	11	111
alpha band	1	1
alpha coherence values	1	2
alpha erd	1	1
alpha event-relateddesynchronization	1	1
alpha power	1	2

alpha power density	2	7
alpha power difference	1	1
alpha range	1	1
alpha rhythms	1	2
alpha sub-bands	2	4
baseline alpha rhythms	1	1
cortical alpha rhythms	2	2
determination ofthe alpha sub-bands	2	4
different alpha band rhythms	2	2
frontal alpha	1	3
frontoparietal alpha	1	2
highfrequency alpha	1	1
high-frequency alpha	1	2
highfrequency alpha band	1	1
high-frequency alpha band	5	7
high-frequency alpha erdwas	1	2
high-frequency alpha rhythms	5	10
highfrequency alpha sub-bands	1	3
individual alpha	1	1
individual alpha frequency	4	4
individual alpha frequency peak	1	1
interpolated alpha	1	1
low frequency alpha rhythms	1	2
low-amplitude alpha	1	2
lowfrequency alpha band	1	1
low-frequency alpha band	5	8
lowfrequency alpha rhythms	2	2
low-frequency alpha rhythms	4	5
occipital alpha power	2	4
ofdominant alpha rhythms	1	1
ofindividual alpha	1	1
parietal alpha	1	2
present low-amplitude alpha	1	1
previous alpha erd studies	1	2
pronounced alpha	4	4
relative alpha	1	1
resting alpha rhythms	1	1
sensorimotor alpha rhythms	1	2
significant alpha	1	1
theamplitude oflow-frequency alpha	1	1
topographic mapping ofthe alpha	1	1
analysis	15	67
analysis ofalpha rhythms	1	1
analysis ofeach piece ofdata	1	2
analysis ofthe artifact-free	1	1

analysis ofthe artifact-free eeg data was based on fft approach	1	1
artifact analysis	1	1
control correlation analysis	1	1
correlation analysis	2	2
correlational analysis	1	1
cortical source analysis	2	3
cortical source analysis ofthe	1	2
final analysis	1	1
first level analysis	1	1
first-level analysis	1	1
group analysis	3	4
individual analysis	2	2
measure analysis	1	1
nonparametric correlation analysis	1	2
parameter analysis	1	1
power spectrum analysis	2	2
pragmatic analysis	1	1
preliminary data analysis	2	2
present control analysis	1	1
principal component analysis	1	1
random effects analysis	1	1
random-effects group analysis	1	1
repeated measures analysis	1	2
second-level analysis	1	1
second-level group analysis	1	2
seedto-voxel-based analysis	1	1
seed-to-voxel-based analysis	1	1
semantic analysis	1	1
session analysis	1	3
single-session analysis	1	1
spectral analysis	1	1
stabilometric data analysis	1	3
statistical analysis	8	10
stimulus type analysis	1	2
two-way analysis	1	1
whole brain analysis	1	1
area	1	7
cerebellar areas	1	1
contralateral sensorimotor area	1	2
cortical foot area	1	1
hand area	1	1
temporal areas	1	1
ventral premotor areas	1	1
areas	15	78

brodmann areas	1	2
centroparietal area	1	1
cerebellar areas	2	3
certain brain areas	1	1
cingulate motor area	1	1
contralateral sensorimotor area	3	6
core areas	1	1
cortex areas	1	1
cortical areas	1	2
cortical foot area	1	1
cortical hand area	1	1
critical brain areas	1	1
dorsolateral pre-motor area	1	2
following brain areas	1	2
hand area	2	2
innovative areas	1	1
ipsilateral sensorimotor area	1	2
local areas	1	1
mean area	2	2
medial premotor areas	2	2
motor-related areas	1	1
neural areas	1	1
occipital areas	2	5
orbitofrontal area	1	1
parahippocampal area	1	1
parietal areas	3	4
parietal-occipital areas	1	1
prefrontal areas	1	1
premotor areas	1	1
primary motor areas	4	5
secondary motor areas	1	1
somatomotor area	1	1
supplementary motor area	5	8
sway area	1	3
taskrelevant brain areas	1	2
task-relevant brain areas	1	1
task-sensitive areas	1	1
temporal areas	2	2
temporoparietal area	1	1
trunk area	1	1
ventral premotor areas	1	1
athletes	14	77
14 table tennis athletes	1	1
18 elite karate athletes	1	3
35 novice athletes	1	1

amateur athletes	2	2
american football athletes	1	1
athlete condition	1	2
athlete group	1	1
athlete iaps	1	1
athlete training	1	1
athletes objective	1	1
badminton athletes	1	1
baseball athletes	1	1
college athletes	1	1
disabled athletes	1	1
elite athletes	6	15
elite basketball athletes	2	2
elite fencing athletes	1	3
élite golf athletes	1	1
elite karate athletes	3	10
experienced athletes	1	1
expert athletes	1	2
expert basketball athletes	1	1
fencing athletes	2	2
gymnastic athletes	2	2
high-level athlete	1	1
involving athletes	1	1
karate athletes	3	6
male athletes	1	1
non athletes	1	1
present athletes	2	2
professional karate athletes	1	2
prompt athletes	1	1
skilled athletes	2	2
table tennis athletes	1	1
type athletes	1	1
young overhead athletes	1	1
attention	13	35
attention control	1	1
attentional cueing task	1	3
attentional load condition	1	3
attentional load levels	1	1
attentional processes	3	3
attentional resource allocation	1	2
dorsal attention network	1	1
encompassing attentional	1	1
enhanced attention	1	1
global attentional	2	2
goal-driven attention	1	1

little attention	1	1
orienting attention	1	2
paying attention	3	3
selective attention	1	1
size attention effect	1	1
skillfocused attention	1	1
spatial attention	1	1
special attention	4	4
visuospatial attention	2	2
brain	14	92
3 brain circuits	1	1
31 brain slices	1	1
alert brain information	2	2
anatomic brain imaging	1	2
bilateral brain regions	1	1
brain activation	3	3
brain activation pattern	1	1
brain activity	6	9
brain compartments	3	6
brain damage	1	1
brain electromagnetic activity	1	1
brain information processing	2	2
brain plastic adaptation	1	1
brain plasticity	1	1
brain processes	1	1
brain regions	3	3
brain resources	1	1
brain rhythmicity	1	1
brain shape	1	1
brain structures	1	1
certain brain areas	1	1
critical brain areas	1	1
defining brain correlates	1	1
event-related brain potentials	1	1
extended brain networks	1	1
following brain areas	1	2
frontoparietal brain regions	1	2
functional brain feature	1	1
functional brain networks	1	2
fundamental capability ofhis football brain	1	1
global brain activity	1	1
global brain arousal	1	1
human brain	1	1
investigating brain activation	1	2
low-resolution brain	3	4

main brain circuits	1	2
noninvasive brain stimulation	1	1
normalized brain	1	2
previous brain activity studies	1	2
reduced brain activation	1	1
scanned brain activity	1	1
significant brain regions	1	1
specific brain activation	1	1
standardized brain space	1	1
structural brain variation	2	2
talairach probability brain atlas	3	3
task-related brain regions	1	1
taskrelevant brain areas	1	2
task-relevant brain areas	1	1
understanding brain plasticity	1	1
whole brain	3	3
whole brain analysis	1	1
whole brain measure	1	2
whole brain results	1	2
cognitive	14	68
adopted cognitive tasks	1	1
cognitive aspects	1	1
cognitive control regions	1	1
cognitive demands	1	1
cognitive disruption	1	1
cognitive effort	1	2
cognitive experience	1	1
cognitive information	3	3
cognitive information processing	1	1
cognitive measures	1	1
cognitive performance	4	4
cognitive processes	1	1
cognitive reappraisal	1	1
cognitive representations	1	1
cognitive resources	1	2
cognitive responses	1	1
cognitive tasks	10	20
cognitive test	1	1
domain-specific cognitive	1	1
domain-specific cognitive skills	1	1
enabled cognitive skills	1	1
extensive cognitive test battery	1	1
general cognitive domain owing	1	2
involving cognitive control	1	1
irrelevant cognitive processes	1	1

low cognitive performance	1	1
multidimensional cognitive profile	1	1
overlapping cognitive processes	1	2
performing cognitive	1	1
probing cognitive functions	4	4
several cognitive	1	1
sportrelated cognitive strategy	1	1
substantial cognitive sources	1	1
training-related cognitive	1	1
transferable cognitive benefits	1	1
using cognitive paradigms	1	1
visuospatial cognitive task performance	1	1
conditions	13	52
active tracking conditions	1	1
athlete condition	1	2
attentional load condition	1	3
backswing skill condition	1	1
baseline bipodalic condition	1	2
certain conditions	4	4
closed conditions	1	1
different conditions	4	5
easy conditions	1	1
experimental conditions	1	1
eyesclosed condition	1	1
eyes-closed condition	1	3
eyes-open conditions	1	3
factor condition	2	2
four-target conditions	1	1
invalid condition	1	1
low shot skill condition	1	2
monopodalic conditions	1	3
neutral condition	1	1
open conditions	1	2
passive viewing condition	1	2
peculiar conditions	1	1
rest condition	1	1
resting state condition	1	1
specified conditions	4	4
stressful conditions	1	1
unrelated condition	1	1
valid condition	1	1
control	14	63
agematched control group	1	2
athletic controls	1	8
attention control	1	1

cognitive control regions	1	1
control analyses	1	1
control comparison	1	2
control contrast	1	1
control correlation analysis	1	1
control experiment	2	3
control group	2	2
control hypothesis	3	3
control hypothesis relative	1	1
control iaps	1	1
control participants	1	1
control subjects	5	9
control systems	1	1
control variable	1	1
efficient foot motor control	2	2
efficient foot motor control	2	2
efficient motor control	1	3
executive control network	1	1
extensive executive control activities	1	1
inhibitory control ability	1	1
involving cognitive control	1	1
matched controls	1	1
naïve control	1	1
nonathlete controls	1	1
nonathletic controls	1	2
possible control capacity	1	1
present control analysis	1	1
sex-matched control non-players	1	2
smooth control	1	1
sophisticated muscle synergy control	1	1
static control	1	1
voluntary motor control	1	1
cortex	13	46
bilateral premotor cortex	1	1
cerebral cortex	1	3
cingulate cortex	1	1
cortex areas	1	1
cuneal cortex	1	1
extrastriate cortex	1	2
frontal cortex	2	2
gyrus premotor cortex	1	1
inferior temporal cortex	1	2
lateral dorsal premotor cortex	1	1
motor cortex	1	1
motor cortex frompre	1	2

ofsensorimotor cortex	1	1
parietal cortex	4	5
prefrontal cortex	4	10
premotor cortex	2	2
primary motor cortex	4	5
sensorimotor cortex	1	1
somatosensory cortex	2	2
temporal cortex	1	1
ventrolateral pre-motor cortex	1	1
cortical source	1	5
cortical source analysis	1	2
cortical source patterns	1	2
temporal pattern	1	1
data	13	37
accuracy data	1	2
anatomical data	1	1
behavioral data	3	3
bipolar electrooculographic data	3	3
cinematic data	1	1
conflicting data	1	1
data acquisition	1	1
data relative	1	1
demographic data	1	1
dynamic data –	1	2
electroencephalographic data	1	1
electromyographic data	1	1
fmri data	1	1
functional data	1	1
imaging data	1	1
logical data	1	1
magnetic resonance data	3	3
motor imagery data	1	1
neuroimaging data	2	2
physiological data	1	1
preliminary data analysis	2	2
recording ofbipolar electrooculographic data	2	2
stabilogram data	1	1
stabilometric data analysis	1	3
differences	14	51
accuracy difference	1	2
affective personality differences	1	1
alpha power difference	1	1
assessing group differences	1	2
between-groups differences	1	1
expertise-based differences	1	1

functional connectivity differences	1	3
group differences	1	1
group differences	2	3
individual differences	1	1
individual differences	3	4
mean differences	1	1
neural differences	1	1
nonexpert differences	1	1
performance differences	1	1
potential differences	1	1
quantitative differences	1	1
showing differences	1	1
significant differences	6	13
significant differences	2	3
significant inter-group difference	1	1
significant inter-groups difference	2	2
small differences	5	5
effect	12	34
adaptation effect	1	1
anxiolytic effects	1	1
ceiling effect	1	1
cueing effect	1	1
deleterious effects	1	1
effective motor task execution	1	1
effective processing	1	1
exact effect	1	1
interaction effect	1	1
local peak effect	1	1
main effect	4	6
main effect	2	2
mean effect size	1	1
over-years motor skill training effect	1	3
possible effect	1	1
practice effects	1	1
selective expertise effect	1	1
significant effect	1	1
similar training effect	1	1
size attention effect	1	1
slight effects	2	2
statistical effects	2	3
stimulus type effect	1	1
elite	12	56
18 elite karate athletes	1	3
distinguishing elite	1	1
elite archers	3	5

elite athletes	6	15
elite basketball athletes	2	2
elite fencing	1	1
elite fencing athletes	1	3
elite group	1	2
elite gymnasts	1	1
elite karate	2	2
elite karate athletes	3	10
elite kendo	2	2
elite rhythmic	1	1
elite rhythmic gymnasts	1	1
elite rifle	2	2
elite rugby players	1	1
elite shooters	1	1
elite table tennis players	3	3
error	1	3
low judgment error	1	1
mean judgment error	1	1
zero standard error	1	1
expert	13	37
archery experts	1	1
comparing experts	1	2
expert advantage	1	1
expert archers	1	1
expert athletes	1	2
expert basketball athletes	1	1
expert behavior	1	1
expert dancers	3	4
expert electroencephalographists	3	3
expert groups	1	2
expert guitarists	1	1
expert judgments	1	1
expert motor performance	1	2
expert performance approach	1	2
expert performers rules	1	1
expert pianists	1	1
expert pistol shooters	2	2
football expert	1	1
golf experts	2	4
non expert	1	1
studying experts	1	1
tennis table experts	1	2
football	4	29
13 football players	1	1
amateur football tournaments	1	1

american football athletes	1	1
brasilian footballer	1	2
football competition	1	1
football experience	2	4
football expert	1	1
football game	1	1
football scenes	1	1
football skills	1	1
fundamental capability ofhis football brain	1	1
joined football clubs	1	1
male football	1	1
paradigmatic football action	1	2
playing football	1	1
practicing football	1	3
pro footballers	1	3
professional football coach	1	1
professional footballers	1	1
skillful footballer	1	1
frontal	8	31
frontal alpha	1	3
frontal anticipatory	1	1
frontal cortex	2	2
frontal cortices	1	1
frontal eye field	1	1
frontal eye fields	1	1
frontal gyrus	2	2
frontal karate punches	1	1
frontal mirror system	1	2
frontal pole	1	1
frontal punches	1	1
frontal regions	2	4
inferior frontal gyrus	2	4
medial frontal gyrus	1	2
middle frontal gyrus	1	1
motor frontal	1	1
right frontal	1	1
superior frontal gyrus	1	2
functional	14	66
adaptive functions	1	1
cortical function	6	6
executive functioning	1	1
functional brain feature	1	1
functional brain networks	1	2
functional changes	1	1
functional connectivity differences	1	3

functional coupling	1	1
functional data	1	1
functional equivalence	1	1
functional feature	1	1
functional images	5	8
functional imaging technique	3	3
functional links	1	1
functional measure	1	1
functional modes	4	4
functional organization	1	1
functional pattern	1	1
functional reorganization	1	2
functional status	1	1
functional volumes	2	3
hanning function	1	1
hemodynamic response function	2	3
interpolating function	1	1
probing cognitive functions	4	4
spline function	4	4
spline interpolating function	2	2
trunk function	1	1
trunk muscle function	1	1
windowing function	5	5
group	9	34
agematched control group	1	2
anticorrelation group	1	1
assessing group differences	1	2
athlete group	1	1
certain group	1	1
control group	2	2
elite group	1	2
expert groups	1	2
factors group	1	1
group analysis	3	4
group comparisons	1	1
group diﬀerences	1	1
group differences	2	3
group statistics	1	1
karate group	1	4
participant groups	1	1
random-effects group analysis	1	1
second-level group analysis	1	2
undifferentiated group self report	1	2
gymnastic	1	8
gymnastic athletes	1	1

gymnastic performances	1	2
gymnastic referees	1	1
gymnastic videos	1	2
rhythmic gymnastics	1	2
gymnasts	1	4
non gymnasts	1	1
rhythmic gymnasts	1	3
gyrus	7	50
angular gyrus	1	1
bilateral parahippocampal gyrus	1	2
cingulate gyrus	1	1
frontal gyrus	2	2
fusiform gyrus	1	2
gyrus post	1	2
gyrus posterior	1	1
gyrus premotor cortex	1	1
inferior frontal gyrus	2	4
inferior temporal gyrus	2	2
lingual gyrus	1	1
lingual gyrus thalamus	1	2
medial frontal gyrus	1	2
middle frontal gyrus	1	1
middle temporal gyrus	2	2
middle temporal gyrus ant connections	1	3
occipital gyrus	1	2
parahippocampal gyrus	1	1
postcentral gyrus	4	7
posterior cingulate gyrus	1	1
precentral gyrus	1	1
superior frontal gyrus	1	2
superior temporal gyrus	2	3
supramarginal gyrus	2	2
temporal gyrus	1	2
high-frequency alpha	1	6
high-frequency alpha band	1	1
high-frequency alpha erdwas	1	2
high-frequency alpha rhythms	1	3
images	14	75
48 sport images	1	1
60 volume images	1	1
anatomic brain imaging	1	2
anatomical image	3	4
contrast images	2	2
diffusor tensor imaging	1	1
echo-planar imaging	1	1

emotion-eliciting images	1	1
functional images	5	8
functional imaging technique	3	3
generalized nonsport images	1	2
image acquisition time	1	1
image sets	1	1
image size	1	1
image type	1	1
imaging data	1	1
individual cluster image	1	2
kinesthetic images	1	1
magnetic resonance imaging	13	17
magnetic resonance imaging study	1	2
middle image	1	1
negative images	1	3
neutral images	1	2
normalized images	1	1
planar imaging	2	2
planar imaging sequence	2	2
preprocessed images	1	1
sensory image representations	1	1
static images	2	2
structural images	1	1
template images	1	1
time-corrected image	1	1
unpleasant images	1	1
weighted echo-planar images	1	1
weighted images	2	2
imaging	1	3
functional imaging technique	1	1
magnetic resonance imaging	1	1
static images	1	1
information	11	63
advanced information	1	1
alert brain information	2	2
brain information processing	2	2
cognitive information	3	3
cognitive information processing	1	1
color information	1	1
cortical information processing	3	3
extracting information	1	1
information processing	2	3
internal information	1	1
next information integration	1	1
ofspatial information contents	1	1

processing ofvisuo-spatial andmotor information	1	2
related information processing	1	1
related stimulus information	1	2
semantic information	3	3
sensorimotor information	1	2
sensorimotor information flows	1	1
sensory information	3	3
sequential information	1	1
spatial information	1	1
spatial information content	3	4
sportspecific information	1	1
three-dimension information	1	1
visual information	1	1
visual information elaboration	1	1
visual information flow	1	1
visual information flux	2	6
visual information processing	2	4
visuo-spatial information	1	1
visuospatial information processing	1	5
visuo-spatial information processing	1	1
whole information processing flow	1	1
inverse	1	4
inverse problem	1	1
linear inverse algorithms	1	1
linear inverse solution procedures	1	2
judgment	1	10
action judgment	1	2
good judgment	1	1
judgment ofsportingobservedactions	1	1
judgment scores	1	2
judgment value	1	1
low judgment error	1	1
mean judgment error	1	1
technical judgments	1	1
karate	8	62
18 elite karate athletes	1	3
contrasting karate	1	1
elite karate	2	2
elite karate athletes	3	10
erroneous karate performance	1	1
extensive karate training	1	2
frontal karate punches	1	1
italian karate team	1	1
judgmentofobserved karate actions	1	1
karate actions	2	2

karate athletes	3	6
karate attacks	3	3
karate discipline context	1	2
karate experience	1	1
karate exposure	1	1
karate group	1	4
karate picture	1	1
karate program	1	1
karate referees	1	1
karate repertoire	1	1
karate stimuli	1	1
karate videos	2	4
low-level karate performances	1	1
national karate	1	1
national karate team	1	1
ofambiguous karate performances	1	1
played karate	1	2
practicing karate	2	3
professional karate athletes	1	2
typical karate position	1	1
level	13	34
2nd level	1	1
agonistic level	1	2
amateur level	1	1
athletic level	2	2
attentional load levels	1	1
behavioral level	1	1
blood-oxygen level	1	2
cluster level	3	4
college level	1	1
competitive level	1	3
different levels	1	1
eye level	1	1
first level analysis	1	1
graded levels	1	1
low levels	1	1
national level	1	1
neural level	1	1
professional level	1	1
significant level	1	1
skill level	1	3
spanish national-training-center level swimmers	1	1
stable level	1	1
voxelwise threshold level	1	1
whole-brain level	1	1

mean	11	32
mean ambiguity index	1	2
mean area	2	2
mean center	1	1
mean contrast values	2	3
mean differences	1	1
mean effect size	1	1
mean judgment error	1	1
mean luminance	1	2
mean number	4	4
mean percentage changes	1	1
mean rating	1	1
mean score	1	1
mean subjects	5	8
mean value	1	1
mean voltage	1	1
using means	1	1
wasintroducedtothe meaning	1	1
mean luminance	1	2
mean luminance	1	2
model	14	29
animal models	1	2
appropriate model	1	1
cortical model	2	2
dipole modeling	3	3
factorial model	1	1
forward models	1	1
head modeling procedures	1	1
internal models	1	1
linear model	3	4
model perspective	1	1
neural efficiency model	1	1
scalp surface model	1	1
speculative model	1	1
spherical head model	3	6
template model	1	2
theoretical model	1	1
motor	15	140
acquired motor skills	1	1
appropriate motor response	1	1
ballistic motor skills	1	1
central motor representation	1	1
central motor system	1	1
cingulate motor area	1	1
complex motor tasks	1	1

cortical motor	1	1
cortical motor systems	1	1
effective motor task execution	1	1
efficient foot motor control	2	2
efficient foot motor control	2	2
efficient motor control	1	3
expert motor performance	1	2
finger motor skills	1	1
first-person perspective motor imagery	1	5
following motor imagery task	1	3
foot motor skills	1	1
general motor imagery ability	1	1
internal motor representation	1	1
left medial-wall foot motor regions	1	3
measuring motor reactivity	1	1
medial-wall motor region	1	1
mirror motor neurons	1	1
mostly motor	1	1
motor abilities	1	1
motor act	2	2
motor action	1	2
motor category	1	1
motor commands	1	1
motor cortex	1	1
motor cortex frompre	1	2
motor domain	1	1
motor execution	2	6
motor frontal	1	1
motor imagery data	1	1
motor imagery paradigm	1	1
motor imagery time	1	1
motor imagery trials	1	1
motor intentions	1	1
motor output	1	1
motor performance	1	1
motor planning regions	1	1
motor potential	1	3
motor practice	1	1
motor preparation	4	4
motor regions	1	1
motor repertoire	2	2
motor representation	1	1
motor response	3	5
motor sequence	1	1
motor sequence task	1	2

motor simulations	1	1
motor skill abilities	1	1
motor systems	3	4
motor tasks	6	9
novel hand motor skill	1	1
novice motor performance	1	2
over-years motor skill training effect	1	3
performing motor tasks	1	2
predictive motor plan	1	2
primary motor areas	4	5
primary motor cortex	4	5
prolonged motor experience	1	1
reactive motor performance	1	1
refined motor skills	1	1
secondary motor areas	1	1
sequential motor behaviors	1	1
skilled motor	1	1
supplementary motor	2	2
supplementary motor area	5	8
trained motor tasks	1	1
untrained motor acts	1	2
voluntary motor control	1	1
well-developed motor skills	1	1
movements	14	105
arm movement	1	1
attacking move	1	1
average movement distance	1	4
ballistic movements	1	2
body movements	1	1
complex finger movements	2	2
dance movements	1	1
decomposed movements	1	1
different movements	1	1
eye movement correction	1	2
eye movements	5	8
finger movements	3	4
global movement	1	1
goal-directed movement tasks	1	1
gross movements	1	1
hand movements	2	2
head movements	4	4
highly-patterned movements	1	1
imagining movements	1	1
intentional movements	1	1
involuntary mirror movements	2	3

kinesthetic movements	1	1
left movement	1	7
movement directions	1	1
movement onset	2	3
movement planning	1	1
movement side	1	1
moving period	1	1
multi-joint body movements	1	1
next move	1	1
nonpracticed movements	1	1
observed dancing movements	1	1
ofnonpracticed movements	1	1
overt movements	1	1
performance movement	1	1
preparation ofthe movements	1	1
psychoergonomic movement parameters	1	3
recognized dancing movements	1	1
recognized movements	1	1
rehearsing movements	1	1
right finger movements	1	1
right foot movements	1	2
right hand movements	1	1
right movements	1	7
saccadic eye movements	1	2
self-paced finger movements	2	2
shoulder movements	1	1
simple foot movement task	1	2
simulated movements	1	1
simultaneous movements	1	1
small movements	1	1
total movement distance	1	1
triggered one-digit movements	1	1
trunk movements	1	1
typical movements	1	1
unilateral hand movements	1	1
viewed movements	2	2
voluntary hand movements	2	2
voluntary movement	2	3
neural	15	101
cortical neural efficiency	1	1
neural activation patterns	1	1
neural activity	7	12
neural activity changes	1	2
neural areas	1	1
neural circuits	1	1

neural cognitive-motor	1	1
neural correlates	3	7
neural cost	1	1
neural differences	1	1
neural dynamics	1	1
neural efficacy	1	1
neural efficiency	1	4
neural efficiency	1	1
neural efficiency	4	19
neural efficiency hypotheses	1	2
neural efficiency hypothesis	2	4
neural efficiency hypothesis	1	1
neural efficiency mode	1	4
neural efficiency model	1	1
neural effort	1	1
neural level	1	1
neural mechanisms	5	5
neural networks	5	8
neural populations	1	1
neural processes	3	4
neural proficiency hypothesis claims	1	1
neural resources	1	4
neural source	1	1
neural substrates	2	3
neural systems	4	4
neural underpinnings	2	2
neural efficiency	1	8
neural efficiency	1	8
ofthe	9	26
analysis ofthe artifact-free	1	1
analysis ofthe artifact-free eegdatawasbasedonfftapproach	1	1
cortical source analysis ofthe	1	2
determination ofthe alpha sub-bands	2	4
early phase ofthe	1	1
efficacy ofthe gesture	1	1
left-down angle ofthe computer keyboard	1	1
local ethics committee ofthe	1	1
negative value ofthe	1	1
ofthe mrps	1	1
ofthe red	1	1
ofthe video presentation	1	1
order ofthe recording blocks	1	1
piece ofthe present results	1	2
preparation ofthe movements	1	1
processing ofthe targets	1	1

recorded amplitude ofthe subject	1	1
right side ofthe monitor	1	2
surface ofimpact ofthe plank	1	1
topographic mapping ofthe alpha	1	1
pathway	1	7
dorsal pathway	1	2
ventral pathway	1	3
visual pathway	1	2
performance	13	87
actual performance	1	1
athletic performance	3	4
behavioral performance	4	8
behavioral task performance	1	1
behavioural performance	1	1
cognitive performance	4	4
cognitive-motor performance	1	1
competitive performance	2	2
correcting performance parameters	1	1
dancing performance	1	1
different performance	1	1
erroneous karate performance	1	1
eventual performance	1	1
experienced performance	1	1
expert motor performance	1	2
expert performance approach	1	2
gymnastic performances	2	3
high-level performance	1	1
kinematic performances	1	2
low cognitive performance	1	1
low-level karate performances	1	1
mot performance	1	1
motor performance	1	1
multiple object tracking performance	1	4
multiple-object tracking task performance	1	2
novice motor performance	1	2
ofambiguous karate performances	1	1
optimal performance	1	2
optimal-controlled performance states	1	1
performance differ	1	2
performance differences	1	1
performance movement	1	1
performance problems	1	1
performance scores	1	1
physical performance	1	1
physical performance output	1	1

reactive motor performance	1	1
real competition performances	1	1
reproducing sporting performances	1	1
shooting performances	1	2
skilled performance	1	1
sports performance	2	3
superior performance	4	5
superior sport performance	1	1
task performance	3	4
visual search reaction time performance	1	4
visuo-motor performance	1	1
visuospatial cognitive task performance	1	1
players	14	35
13 football players	1	1
14 table tennis players	1	2
23 basketball players	1	2
active players	1	1
amusician playing	1	1
baseball players	1	1
dejected players	1	1
elite rugby players	1	1
elite table tennis players	3	3
experience playing	1	1
inexperienced soccer players	1	1
injured player	1	1
keyboard players	1	1
male badminton players	1	2
played karate	1	2
playing football	1	1
playing imitation guitar chords	1	1
professional piano players	2	2
professional player	1	1
skilled players	1	1
soccer players	2	2
table tennis players	1	1
test badminton players	1	1
top-level badminton players	1	1
volleyball players	1	2
wheelchair basketball player classification	1	1
potential	9	34
averaged scalp potentials	2	3
cerebral potentials	1	1
event-related brain potentials	1	1
event-related potentials	1	1
event-related potentials activity	1	2

evoked potentials	1	1
excitatory postsynaptic potentials	1	1
low-amplitude potential shifts	1	1
motor potential	1	3
movement-related potentials	1	1
negative potential	1	1
potential confounds	1	1
potential differences	1	1
potential distribution	2	2
potential influences	1	1
potential lead field	3	3
potential position	1	1
potential relevance	1	1
readiness potential	4	5
scalp potentials	1	1
slow potentials	2	2
power	11	42
alpha power	1	2
alpha power density	2	7
alpha power difference	1	1
eventrelated power decrease	1	2
event-related power decrease	3	4
explanatory power	1	1
field powers	1	1
global source power	1	1
instant power density	1	1
maximal aerobic power	1	1
maximum power	2	2
maximum power density peak	1	2
occipital alpha power	2	4
percentage ratio ofalpha power	1	2
power decrease	1	1
power decrease ofalpha	1	1
power density	1	2
power density spectrum	1	1
power indexes	1	1
power spectra	1	1
power spectrum analysis	2	2
representative power factor	1	1
statistical power	1	1
present	12	39
always present	3	3
piece ofthe present results	1	2
present approach	1	1
present athletes	2	2

present control analysis	1	1
present finding	1	1
present findings	2	2
present high-resolution	1	1
present low-amplitude	1	1
present low-amplitude alpha	1	1
present mrps	1	1
present procedure	1	1
present results	4	5
present stage	1	1
present study	8	16
procedure	12	29
automatic procedure	1	1
auto-regressive procedure	1	1
computerized procedure	2	2
experimental procedure	8	9
following procedure	1	1
geisser procedure	2	2
head modeling procedures	1	1
included procedures	1	1
last response selection procedure	1	1
linear inverse solution procedures	3	6
preprocessing procedure	1	1
present procedure	1	1
realignment procedure	1	1
task procedure	1	1
procedures	1	4
computerized procedure	1	1
experimental procedure	1	1
linear inverse solution procedures	1	2
processing	12	76
adaptive processing	1	1
attentional processes	3	3
auditory affective processing	1	1
basic processing speed	1	1
brain information processing	2	2
brain processes	1	1
cognitive information processing	1	1
cognitive processes	1	1
complex processes	1	1
cortical information processing	3	3
decision-making processes	1	1
deliberate processes	1	1
early processing	1	1
effective processing	1	1

efficient processing	1	1
effortful processing	1	1
effortful processing	1	1
emotional processing	1	2
emotive processes	1	1
execution process	1	1
information processing	2	3
internal processes	1	1
irrelevant cognitive processes	1	1
memory processes	1	1
memory retrieval processes	1	1
mental process	1	1
neural processes	3	4
overlapping cognitive processes	1	2
perceptual processing	1	1
processing ofthe targets	1	1
processing ofvisuo-spatial andmotor information	1	2
processing sport-specific advance cues	1	2
rapid processing	1	1
related information processing	1	1
resting-state processing	1	1
sensorimotor processes	2	2
sensory processing	2	2
similar processes	1	1
speech sound processing	1	1
subsequent processing	1	1
task preparation processes	1	1
task-irrelevant processes	1	1
task-relevant processing	1	1
various processes	1	1
verbal-analytical processing	1	1
visual information processing	2	4
visual processes	1	1
visuospatial information processing	1	5
visuo-spatial information processing	1	1
visuospatial processes	2	3
whole information processing flow	1	1
rate	11	33
accuracy rates	1	1
acquisition rate	1	1
affective ratings	1	2
behavioral ratings	1	1
clinician ratings	1	1
false detection rate	1	1
false discovery rate	1	1

false discovery rate correction	1	2
frequency rate	2	2
mean rating	1	1
rated sport pictures	1	1
rating approach	1	1
rating instructions	1	1
rating period	1	1
rating selection	1	1
sampling rate	7	15
regions	7	32
bilateral brain regions	1	1
border regions	1	1
brain regions	3	3
cognitive control regions	1	1
cortical regions	2	3
critical regions	1	1
emotive regions	1	1
frontal regions	2	4
frontoparietal brain regions	1	2
left medial-wall foot motor regions	1	3
medial-wall motor region	1	1
medial-wall motor-cortical foot regions	1	2
medial-wall regions	1	1
motor planning regions	1	1
motor regions	1	1
occipital regions	1	2
prefrontal regions	1	1
significant brain regions	1	1
subcortical regions	1	1
task-related brain regions	1	1
response	10	49
accurate response	1	1
adaptive response	1	1
affective responses	1	1
appropriate motor response	1	1
behavior responses	1	1
behavioral responses frontiers	1	2
cerebral responses	1	3
cognitive responses	1	1
coping responses	1	1
correct responses	2	4
cortical responses	1	1
dependent response	1	1
discriminative response task	1	1
efficient response strategy	1	1

emotional responses	1	3
fast response	1	1
hemodynamic response function	2	3
hypothalamic response	1	1
key response	1	1
last response selection procedure	1	1
maximum response time	1	1
motor response	3	5
mri-compatible response device	1	1
perturbed response	1	1
response accuracy	1	1
response execution	2	2
response inhibition	1	1
response oftheoperanthand	1	1
response selection	1	1
response selection ability	1	1
response time	1	1
simple response	1	1
transient response time	1	2
rhythmic	1	7
brain rhythmicity	1	1
elite rhythmic	1	1
rhythmic gymnastics	1	2
rhythmic gymnasts	1	3
rhythms	9	35
alpha rhythms	1	2
analysis ofalpha rhythms	1	1
baseline alpha rhythms	1	1
beta rhythms	1	1
cortical alpha rhythms	2	2
different alpha band rhythms	2	2
high-frequency alpha rhythms	5	10
low frequency alpha rhythms	1	2
lowfrequency alpha rhythms	2	2
low-frequency alpha rhythms	4	5
modulation ofalpha rhythms	1	1
mu rhythm	2	2
ofdominant alpha rhythms	1	1
resting alpha rhythms	1	1
sensorimotor alpha rhythms	1	2
right	12	46
right ankle	1	2
right anterior	1	1
right arm	1	1
right attack	1	2

right button	1	1
right cueing	1	1
right eye dominance	1	1
right finger movements	1	1
right foot movements	1	2
right frontal	1	1
right gastrocnemius	1	1
right hand	1	1
right hand movements	1	1
right hemisphere	2	3
right hippocampus	1	1
right index	2	2
right index finger	1	1
right insula	1	4
right key	2	3
right lentiform nucleus	1	1
right movements	1	7
right occipital	1	1
right paracentral lobule	1	1
right precuneus	1	1
right side	1	2
right side ofthe monitor	1	2
superolateral right canthus	1	1
selective	1	4
automatic selection	1	1
selective activation	1	1
selectively active	1	1
spatially selective	1	1
significant interaction	1	4
significant interaction	1	4
single trials	1	5
single trials	1	5
skills	15	60
acquired motor skills	1	1
acquisition ofmotor skills	1	1
anticipation skills	1	1
anticipatory skill	1	1
backswing skill condition	1	1
ballistic motor skills	1	1
domain-specific cognitive skills	1	1
enabled cognitive skills	1	1
equilibrium skills	1	1
finger motor skills	1	1
foot motor skills	1	1
football skills	1	1

low shot skill condition	1	2
motor skill abilities	1	1
novel hand motor skill	1	1
open skill discipline	1	1
open sport skills	1	2
over-years motor skill training effect	1	3
paced skills	1	1
professional skill training	1	1
psychological skills	1	1
psychomotor skills	1	1
refined motor skills	1	1
self-paced skills	1	1
sensorimotor skills	1	1
skill cue types	1	2
skill factor	1	1
skill level	1	3
skill transfer	1	1
skill type	1	2
skilled athletes	2	2
skilled individuals	1	1
skilled marksmen	1	1
skilled motor	1	1
skilled performance	1	1
skilled performers	1	1
skilled players	1	1
spatial skills	8	8
specific skills	4	4
visual perception skills	1	1
well-developed motor skills	1	1
solution	1	5
linear inverse solution procedures	1	2
linear solutions	1	1
sloreta solutions	1	1
weighted minimum norm solution	1	1
sport	14	62
48 sport images	1	1
9 sporting scenes	1	1
closed-skill sports	1	1
competitive sport	2	3
depicting sport situations	3	3
familiar sports	1	1
included sports	1	1
judging sport actions	1	1
observed sporting actions	2	4
ofobserved sporting actions	1	2

open sport skills	1	2
open-skill sports	1	1
peculiar sporting situations	1	1
rated sport pictures	1	1
regular sporting seasons	1	1
reproducing sporting performances	1	1
shooting sports	1	2
situational sports	1	1
specific sport domain	1	1
sporadic sport accidents	1	1
sport activity	1	2
sport anxiety	1	1
sport domain	1	1
sport features	1	1
sport industry	1	1
sport novices	1	1
sport practice	2	2
sport psychology	1	1
sport types	1	2
sporting actions	1	1
sports experience	1	1
sports expertise	2	6
sports frontiers	1	1
sports journalists	2	2
sports performance	2	3
sports science	1	1
sports training	3	3
sports videos	1	1
superior sport performance	1	1
standard	1	3
gold standard	1	2
zero standard error	1	1
statistical	13	36
group statistics	1	1
network-based statistics	1	1
statistical analyses	4	4
statistical analysis	8	10
statistical comparisons	1	1
statistical effects	2	3
statistical interaction	1	1
statistical maps	1	2
statistical parametric	1	1
statistical power	1	1
statistical results	3	3
statistical session	1	1

statistical significance	1	1
statistical support	2	2
statistical threshold	2	2
t statistic maps	1	2
stimulus	6	31
circle target stimulus	1	1
cross-star target stimulus	1	2
imperative stimulus	1	2
non-target stimulus	1	2
related stimulus information	1	2
stimulus attributes	1	1
stimulus deviance	1	1
stimulus discrimination	1	1
stimulus encoding	1	1
stimulus onset	2	3
stimulus presentation	2	3
stimulus tasks	1	1
stimulus type analysis	1	2
stimulus type effect	1	1
target stimulus	2	2
task-relevant stimulus	1	2
unrelated stimulus	1	1
upcoming stimulus	1	1
visual stimulus	1	1
warning stimulus	1	1
study	15	77
behavioral studies	2	3
cross-sectional study	1	1
electroencephalography studies	1	1
electrophysiological study	1	1
entire study	1	1
fmri studies	1	1
future studies	3	3
lesion studies	1	1
magnetic resonance imaging study	1	2
magnetic stimulation study	2	2
neuroimaging studies	8	8
non-human primate study	1	1
pilot study	1	1
present study	8	16
previous alpha erd studies	1	2
previous brain activity studies	1	2
previous fmri studies	1	1
previous neuroimaging studies	3	3
previous study	5	6

prior fmri study	1	1
private study	4	4
recent study	3	3
reviewing studies	1	1
several studies	2	2
studied novice	1	1
study design	1	1
study participants	1	1
study phases	1	1
studying experts	1	1
tomographic study	5	5
subjects	9	35
control subjects	5	9
experimental subjects	1	1
healthy subjects	3	3
individual subjects	1	1
male subjects	1	1
mean subjects	5	8
methods subjects	1	1
naïve subjects	1	2
of152 subjects	1	1
recorded amplitude ofthe subject	1	1
right-handed subjects	2	2
subjective scale	1	2
subjects selection	1	1
watcher subjects	2	2
systems	13	34
action-monitoring system	1	1
central motor system	1	1
control systems	1	1
cortical motor systems	1	1
cortical systems	2	5
frontal mirror system	1	2
fronto-parietal systems	1	1
integrated system	1	1
memory systems	1	2
mirror neuron system	2	2
mirror systems	1	2
motor systems	3	4
neural systems	4	4
projection system	1	1
specificneural systems	1	1
system reference	1	1
visual systems	1	1
visual ventral system	1	1

working memory systems	1	2
task	15	140
action anticipation task	1	1
adopted cognitive tasks	1	1
aiming task	1	4
attentional cueing task	1	3
attention-switching task	2	2
behavioral task performance	1	1
challenging tasks	1	1
choice reaction time tasks	1	2
closed tasks	1	1
cognitive tasks	10	20
cognitive-motor tasks	1	1
complex motor tasks	1	1
decision-making task	1	1
demanding tasks	3	3
different tasks	1	1
digit span task	2	2
discriminative response task	1	1
distal tasks	1	1
domain-general task	1	1
domainspecific task	1	1
effective motor task execution	1	1
expertise-related tasks	1	1
familiar tasks	1	1
following motor imagery task	1	3
goal-directed movement tasks	1	1
identical task	1	1
inhibition task	1	1
mental arithmetic subtraction task	1	1
motor sequence task	1	2
motor tasks	6	9
multiple-object tracking task performance	1	2
no-go tasks	1	1
oddball task	2	2
perceptual-cognitive tasks	1	3
performing motor tasks	1	2
performing wrist extension task	1	1
pitching discrimination task	1	1
proximal tasks	1	1
reaction time tasks	1	2
reasoning tasks	2	2
retrieval short-term memory tasks	3	3
sensorimotor tasks	2	2
serial reaction time task	1	1

short-term memory tasks	1	1
simple foot movement task	1	2
simple tasks	1	1
social cognition task	2	4
sport-specific task	1	1
stimulus tasks	1	1
supporting task execution	1	2
tapping tasks	1	1
task costs	1	1
task difficulty	1	1
task effects	1	1
task features	1	1
task instructions	1	1
task performance	3	4
task preparation processes	1	1
task procedure	1	1
task specific	2	2
trained motor tasks	1	1
transfer tasks	1	1
visual search task	1	1
visual-spatial task	2	2
visuoattentional task	2	2
visuo-attentional task	1	1
visuo-motor tasks	2	2
visuospatial cognitive task performance	1	1
visuospatial task	1	1
visuo-spatial tasks	2	4
working memory task	3	5
test	1	6
matching test	1	1
post-hoc testing	1	1
sentence–picture verification test	1	1
spearman test	1	2
triplet numbers test	1	1
time	14	56
cessation timings	1	1
choice reaction time tasks	1	2
correct-trial reaction times	1	1
echo time	3	4
equal time	1	1
execution timing	2	3
five times aweek	1	1
frame timer	1	1
image acquisition time	1	1
included slice time correction	1	1

including reaction times	1	1
interval time	1	1
maximum response time	1	1
motor imagery time	1	1
perfect timing	1	1
permanence time	1	1
reaction time	2	6
reaction time tasks	1	2
recording time	1	1
repetition time	4	5
response time	1	1
sequential slice timing	1	1
serial reaction time task	1	1
similar time	1	1
slice timing	1	1
slice timing correction	1	1
speed hand reaction time	1	1
time course	1	1
time error	1	1
time periods	2	2
time series	1	1
time window	1	1
timing adjustment	1	1
transient response time	1	2
visual search reaction time performance	1	4
training	12	58
athlete training	1	1
athletic training	1	3
cadence training	1	1
continuous training	1	1
current training	1	1
daily training	1	1
extensive karate training	1	2
field training	1	1
formal table tennis training experience	1	2
hasbeenshownthat training	1	1
indoor strength training	1	1
intensive training	1	1
intensive visuo-motor training	1	1
long-standing training	1	1
long-term field training	1	1
long-term training	3	4
mental calculation training	1	1
novel training strategies	1	1
over-years motor skill training effect	1	3

perceptual training	1	3
physical training	1	1
physical training introduction	1	2
professional badminton training experience	1	1
professional skill training	1	1
professional training	1	2
professional training experience	1	1
scripted training manual	1	2
similar training effect	1	1
specific training	1	1
sports training	3	3
systematic training	1	1
trained motor tasks	1	1
training effects	1	1
training experience	1	1
training hours	1	1
training programs	1	1
training session	1	1
training shapes	1	1
training strategies	1	1
training years	2	2
typical training week	1	1
trials	9	27
about100eegsingle trials	1	1
adjacent trials	1	1
beginning ofeach trial	1	1
example trial	1	1
invalid trials	1	1
motor imagery trials	1	1
neutral trials	1	2
next trial	1	1
sample trial	1	1
single trials	5	12
trial structure	1	2
trial type	1	1
valid trials	1	1
watch trials	1	1
values	11	30
absolute value	1	1
alpha coherence values	1	2
balanced amplitude values	1	2
beta values	1	1
corresponding values	1	1
gender values	4	4
intermediate value	1	1

judgment value	2	2
maximum percentage values	1	1
mean contrast values	2	3
mean value	1	1
negative percentage values	1	2
negative value ofthe	1	1
negative values	1	1
numeric value	1	1
positive percentage values	1	1
positive values	2	2
valuable evidence frontiers	1	2
valuable sources	1	1
ventral	1	5
first ventral	1	1
ventral pathway	1	3
ventral premotor areas	1	1
videos	1	5
end ofeach video	1	1
gymnastic videos	1	2
video presentation	1	1
whole videos duration	1	1

Note:
1 = Babiloni et al. (2009)
2 = Babiloni et al. (2010)
3 = Berti et al. (2019)
4= Costanzo et al. (2016)
5 = DelPercio et al. (2008)
6 = DelPercio et al. (2009)
7 = DelPercio et al. (2011)
8 = DelPercio et al. (2019)
9 = Guo et al. (2017)
10 = Kim et al. (2014)
11 = Natio & Hirose (2014)
12 = Percio et al. (2010)
13 = Qiu et al. (2019)
14 = Wang & Tu (2017)
15 = Wei & Li (2018)
16 = Wei & Li (2017)
17 = Zhang et al. (2019)
