# Supplementary material for: Neural Efficiency in Athletes: A Systematic Review
Source: Front Behav Neurosci. 2021 Aug 5;15:698555. doi: 10.3389/fnbeh.2021.698555 (PMC8374331; doi:10.3389/fnbeh.2021.698555)
Supplement: Supplementary file 2 [file Data_Sheet_2.pdf]

Name	EG Term Used	All Subject Characteristics	Task Protocol Used	Findings	Notes on Limitations	Unique or Novel Aspects
**Babiloni et al. (2009)**	Elite	Right-handed; M(3), F(25)	Subjects observed a series of 120 rhythmic gymnastics videos. The videos showed elite gymnasts executing real exercises with different kinds of apparatus (rope, hoop, ball, ribbon, and clubs) during national or international competitions. The subjects had to judge the artistic/athletic level of the rhythmic gymnastics exercise by a scale ranging from 0 to 10.	The judgment of observed sporting actions is related to low amplitude of alpha ERD, as a possible index of spatially selective cortical activation NEH.	The present EEG data cannot resolve fine functional topographical details in parietal and premotor areas, when compared to PET or fMRI	A “neural efficiency” would occur according to a complex topographic and temporal pattern.
**Babiloni et al. (2010)**	Elite players	Right-handed; M(23), F (26)	The subjects observed a series of 120 karate videos. Each video lasted 8 s with a random inter-stimulus interval ranging from 4.5 to 5.5 s. A central cross was always present as a target for eyes fixation.	The judgment of observed sporting actions is related to relatively less pronounced alpha ERD, as a possible index of spatially selective cortical activation or NEH	Neuroimaging technology	Patterns of “neural efficiency” are modulated by both intensive task-specific training and neuro-feed-back of alpha ERD.
**Berti et al. (2019)**	Professional	Right-handed, M(8), F(6)*	All subjects underwent an extensive cognitive test battery for the identification of individual multidimensional cognitive profile and rs-fMRI scans investigating functional connectivity (FC).	Kinematic performances of athletic feats were sustained by increased positive correlations between subcortical (cerebellum and left thalamus) and cortical (inferior frontal cortex, superior parietal cortex, superior temporal cortex) regions. Athlete's brain can be considered a model of continuous plastic train-related adaptation.	It is difficult to say that differences in functional connectivity results are specific for karate athletes, as athletes of other martial arts have not been tested. Sample size.	Structural brain adaptations following extensive training are sport-specific and usually take place in brain regions associated with the neural processing of sport-specific skills
**Costanzo et al. (2016)**	Athletes	Right-handed; CS, M(25)*	Each participant was cued to passively view for 8 s 24 non-sport negative images and 24 SS images in addition to the passive viewing of 24 neutral images during randomly intermixed trials. Each image was shown only once to a given participant.	Expert performance was accomplished with fewer neuronal resources compared to novices	Cannot preclude the posterior parietal activation is also associated with attention-related processes, as the posterior parietal areas has been shown to be involved in directing spatial attention and in disengaging and maintaining attention to visual and tactile stimuli.	Elite athletes demonstrate neural efficiency in regions critical to self-regulation and less activation in regions involved in emotional response.
**DelPercio et al. (2008)**	Experts	Right-handed; M(15), F(18)	The subjects were presented with a series of 200 different pictures, depicting fencing (50%) or karate (50%) attacks taken during real actions in elite athletes. The task consisted in judging the side (left/right) of the attack. The subjects were requested to minimize eye movements.	The amplitude of the MRPs was higher in the non-athletes than in the athletes, as an index of spatially selective cortical activation NEH.	Not mentioned the participants are neurologically healthy, no other medical problems or any contraindication to the test method	NEH is independent of visual stimuli depicting “peculiar” sport situations.

Name	EG Term Used	All Subject Characteristics	Task Protocol Used	Findings	Notes on Limitations	Unique or Novel Aspects
**DelPercio et al. (2009)**	Elite athletes	Right-handed; M(10), F(12)	The EEG data were spatially enhanced by surface Laplacian estimation. Cortical activity was indexed by task-related power decrease (TRPD) of EEG alpha power (8–12Hz) during monopodalic referenced to bipodalic condition.	The low- and high-frequency alpha TRPD was lower in the athletes than in the non-athletes. Low-frequency alpha TRPD was lower in amplitude in athletes than in the non-athletes. Similarly, the amplitude of high-frequency alpha TRPD was lower in the athletes than in the non-athletes.	Not mentioned the participants are neurologically healthy, no other medical problems or any contraindication to the test method	The present study extends our understanding of the physiological mechanisms at the basis of the NEH for engaging upright standing in elite athletes
**DelPercio et al. (2011)**	Elite	Right-handed; M(20), F(26)	Data were recorded in the elite karate athletes and non-athletes at resting state. All data were digitized in continuous recording mode. The EEG recordings were performed in the late morning. In order to keep constant, the level of vigilance, an experimenter controlled on-line the subject and the EEG traces.	Athletes' brain is characterized by reduced cortical reactivity to eyes opening in the condition of resting state, in line with the NEH.	Not clear on participants' health conditions.	Motivate future research evaluating the extent to which this general functional brain feature is related to heritable trait or intensive visuo-motor training of elite athletes.
**DelPercio et al. (2019)**	Players	Right-handed; M (26)	Data were recorded during the observation of 70 videos of football actions lasting 2.5 s each. The subject kept the left index (non-dominant hand) on a key placed at the left-down angle of the computer keyboard while the right index (dominant hand) was on a key placed at its right-down angle.	A prominent parietal cortical activity related to visuospatial processes during football scenes was greater in the football players over controls in contrast with the NEH.	Not clear on participants' health conditions.	The parietal cortical activity of football players did not show a neural efficiency functioning during the complex visuospatial information processing related to football attacks on goal.
**Guo et al. (2017)**	Experts	Right-handed; M(28)*	Participants were asked to press the left key with right index finger when the circle target stimulus displayed, press the right key with the right third finger when the cross-star target stimulus showed, and instructed not to press key while non-target stimulus displayed. The total number of trials was 256.	Athletes reacted faster than non-athletes during both types of the tasks, and no accuracy difference was found between athletes and non-athletes. Athletes exhibited less brain activation than non-athletes several brain regions. No region was significantly more activated in the athletes than in the non-athletes.	It is possible that subjects having certain basic perceptual-motor skills received positive feedback during their first attempts to practice sports and they became "athletes".	There is neural efficiency in athletes may stem from the long-term training which prompt athletes to develop a focused and efficient organization of task-related neural networks, and possibly task-specific.
**Kim et al. (2014)**	Elite and Experts	Right-handed; M(13), F(27)*	During functional magnetic resonance imaging, 40 elite, expert, or novice archers aimed at a simulated 70-meter- distant target and pushed a button when they mentally released the bowstring.	The more localized neural activity of elite and expert athletes than novices permit greater efficiency in the complex processes subserved by brain regions. Cerebellum is involved in automating simultaneous movements by integrating the sensorimotor memory enabled by greater expertise in self-paced aiming tasks.	Did not account for transient response time in relation to individual differences in the duration of mental strategies	Brain structures are critical to the development of elite-level performance in a closed motor skill that is shedding more light on the neurologic basis of the expert advantage. Similar brain structures were active across skill levels.
**Naito and Hirose (2014)**	Elite	M (7)	Each participant completed two experimental sessions, each of which consisted of 8 runs. The movements were recorded from the view of the bottom of the foot. The bed's width was also recorded for the calibration to quantify the foot movements.	This study found activations in the left medial-wall foot motor regions during the foot movements consistently across all participants.	Inadequate sample size, no expert-novice comparison. Not clear on participants' health conditions.	Elite athletes' brain could allow them to spend neural resources to focus more on cognitive aspects during a football game, such as anticipating/predicting and detecting the actions of other players

Name	EG Term Used	All Subject Characteristics	Task Protocol Used	Findings	Notes on Limitations	Unique or Novel Aspects
**Percio et al. (2010)**	Elite	Right-handed; M (9), F(13),	Subjects observed a series of 120 rhythmic gymnastics videos. The videos showed elite gymnasts executing real exercise with different kinds of apparatus (rope, hoop, ball, ribbon, and clubs) during national or international competitions. Each video lasted 8 s with a random inter-stimulus interval.	During both preparation and execution of the right movements, the low and high-frequency alpha ERD was lower in amplitude in primary motor area, in lateral and medial premotor areas in athletes than in the non-athletes. For the left movement, only the high-frequency alpha ERD during the motor execution was lower in the athletes than in the non-athletes.	Not clear on participants' health conditions.	Compared with non-athletes, elite athletes are characterized by a reduced cortical activation during simple voluntary movement
**Qiu et al. (2019)**	Experts	Right-handed; M(45)	The participants viewed the stimuli via a small mirror located inside the scanner. The experiment contained 96 trials divided into 24 cycles of four trials each (passive viewing, two targets, three targets, and four tar- gets). The trial sequence order within the cycles was random. Participants had 15 s rest (fixation condition) between blocks.	The non-athlete group had greater cortical activation than athletes in several brain areas including the left FEF and bilateral aIPS. Greater activation was seen in the athlete group than in the non-athlete group in the medial superior frontal gyrus (mSFG). MOT performance reflected more efficient brain use during better task performance.	Did not conduct objective assessments of the subject's abilities. No measurements of network properties were undertaken. Not clear on participants' health conditions.	Neural efficiency may be represented by bidirectional alterations encompassing activation of task-relevant brain regions in conjunction with deactivation of other regions to suppress distraction.
**Wang and Tu (2017)**	Athletes	Right-handed; M(32)*	All participants took part in the experimental procedure that included two phases: the first consisted of obtaining their basic information and an aerobic fitness evaluation and the second comprised the cognitive task with EEG recording.	The contingent negative variation amplitude was smaller for the players than for the controls in the condition involving higher uncertainty.	Failed to observe changes in behavioral performance as a function of different skill cues. The level of uncertainty of the action cues was not effectively manipulated.	Explained by the proficient use of cortical resources in athletes for optimal performance. Accordingly, the capability to perform a sport skill to a certain level helps to better process the sport-specific body movement.
**Wei and Li (2018);**	Experts	Right-handed*	The whole task is the action video of different rotating serve of table tennis. The speed of stimulus presentation was fixed at 16 frames /s, with a ratio of 1:1. There were 140 stimulation tasks in total.	The accuracy rate of expert group action recognition was higher than that of the novice group. The low frequency alpha-ERD and the high-frequency alpha-ERD were lower than that of the novice group.	Not clear mention subjects' gender	Athletes motor recognition is decreased in activation of the occipital-parietal visual cortex and mirror system cortex, increased effective functional connectivity in the right hemisphere task brain region, and decreased inefficient functional connectivity in the left hemisphere and inter-hemispheric brain region.
**Wei and Li (2017)**	Experts	Right-handed*	The subjects were required to keep their eyes at a level with the center of the screen at a distance of 60 cm. Target stimuli and non-target stimuli were presented randomly, among which, target stimuli were 60 times and non-target stimuli were 60 times, a total of 120 times.	The ERP results showed the early neural resources in the frontal and parietal cortex were more depleted, indicating that long-term training could improve the consumption efficiency of cerebral neural resources and promote the adaptability of athletes to rapid processing.	Not clear mention subjects' gender	Neural efficiency can be observed in athletes' brain parietal central during simple visual discrimination task.

Name	EG Term Used	All Subject Characteristics	Task Protocol Used	Findings	Notes on Limitations	Unique or Novel Aspects
**Zhang et al. (2019)**	Experts	Right-handed; M(48); CS	The experiment consisted of two runs: imagining a basketball throw with a basketball and imagining a volleyball serve with a volleyball. The order of the two runs was counterbalanced among the participants. Each run lasted 6 min, during which 180 volumes were acquired. Each run consisted of 25 trials.	Better temporal congruence between motor execution and motor imagery and vividness of motor imagery, but lower levels of activation in the left putamen, inferior parietal lobule, supplementary motor area, postcentral gyrus, and the right insula when both groups of athletes imagined movements from their self-sport compared with when they imagined movements from the other sport.	Not clear on participants' health conditions.	Neural efficiency may stem from the long-term training and be task-specific which enabled athletes to develop a focused and efficient organization of task- related neural networks.

EG = Experimental Group; CS = College Student; *Study disclosed participants with normal or corrected vision and no history of mental disorders problems, no metal in body, no tattoos on face, no medicine delivery patch.
