# Supplementary material for: Neural Efficiency in Athletes: A Systematic Review
Source: Front Behav Neurosci. 2021 Aug 5;15:698555. doi: 10.3389/fnbeh.2021.698555 (PMC8374331; doi:10.3389/fnbeh.2021.698555)
Supplement: Supplementary file 3 [file Data_Sheet_3.PDF]

Supplementary File C

Visualization matrix

The co-occurrences map was generated based on the algorithm which applied a similarity measure known as the association strength (Van Eck & Waltman, 2010). This similarity measure was also referred to as "the proximity index or as the probabilistic affinity index" (Van Eck & Waltman, 2010, p .531). According to Van Eck and Waltman (2010), the similarity s_{ij} between two items i and j is calculated as:

$$s_{ij} = \frac{c_{ij}}{w_i w_j}$$

Then, "where c_{ij} denotes the number of co-occurrences of items i and j and where w_i and w_j denote either the total number of occurrences of items i and j or the total number of co-occurrences of these items" (Van Eck & Waltman, 2010, p. 531). It can be shown that "the similarity between items i and j calculated using the above algorithm is proportional to the ratio between on the one hand the observed number of co-occurrences of items i and j and on the other hand the expected number of co-occurrences of items i and j under the assumption that occurrences of items i and j are statistically independent" (Van Eck & Waltman, 2010, p. 531). The researchers referred to Van Eck and Waltman (2010) for an extensive discussion of the advantages of the association strength over other similarity measures, such as the cosine and the Jaccard index.
